# ER Disposal Pathways in Chronic Liver Disease: Protective, Pathogenic, and Potential Therapeutic Targets

**DOI:** 10.3389/fmolb.2021.804097

**Published:** 2022-01-31

**Authors:** Caroline C. Duwaerts, Jessica L. Maiers

**Affiliations:** ^1^ Department of Medicine, University of California, San Francisco, San Francisco, CA, United States; ^2^ Department of Medicine, Indiana University School of Medicine, Indianapolis, IN, United States

**Keywords:** ER associated degradation, ER-phagy, ER-lysosomal degradation, non-alcoholic fatty liver disease, non-alcoholic steatohepatitis, alcoholic liver disease, alpha-1 antitrypsin disease, fibrosis

## Abstract

The endoplasmic reticulum is a central player in liver pathophysiology. Chronic injury to the ER through increased lipid content, alcohol metabolism, or accumulation of misfolded proteins causes ER stress, dysregulated hepatocyte function, inflammation, and worsened disease pathogenesis. A key adaptation of the ER to resolve stress is the removal of excess or misfolded proteins. Degradation of intra-luminal or ER membrane proteins occurs through distinct mechanisms that include ER-associated Degradation (ERAD) and ER-to-lysosome-associated degradation (ERLAD), which includes macro-ER-phagy, micro-ER-phagy, and Atg8/LC-3-dependent vesicular delivery. All three of these processes are critical for removing misfolded or unfolded protein aggregates, and re-establishing ER homeostasis following expansion/stress, which is critical for liver function and adaptation to injury. Despite playing a key role in resolving ER stress, the contribution of these degradative processes to liver physiology and pathophysiology is understudied. Analysis of publicly available datasets from diseased livers revealed that numerous genes involved in ER-related degradative pathways are dysregulated; however, their roles and regulation in disease progression are not well defined. Here we discuss the dynamic regulation of ER-related protein disposal pathways in chronic liver disease and cell-type specific roles, as well as potentially targetable mechanisms for treatment of chronic liver disease.

## Introduction

The rising global prevalence of liver disease necessitates the development of effective strategies to limit disease progression. While numerous drugs and interventions have entered clinical trials, these strategies have been difficult to translate to patient care (Asrani et al., 2019). This is due in large part to the diverse etiologies and mechanisms that underly disease occurrence and progression (Asrani et al., 2019). Metabolic liver diseases, including Alcoholic Liver Disease (ALD) and Non-alcoholic fatty liver disease (NAFLD) are associated with altered proteomes, as are genetic-associated liver diseases such as Alpha-1 antitrypsin deficiency (AATD). While established as a key driver of AATD pathogenesis, a cellular process that often goes overlooked in other forms of chronic liver disease is ER-associated degradative pathways that maintain proteostasis. Also known as ER quality control pathways, these processes are critical for removing misfolded, unfolded, or modified proteins, limiting endoplasmic reticulum (ER) stress, and maintaining cell viability (Maiers and Malhi, 2019; Hetz et al., 2020). Indeed, several lines of evidence suggest that enhanced protein degradation could limit hepatocyte damage and hepatocyte dysfunction, inflammation, and disease progression (Kim and Kim, 2020; Xia et al., 2020; Flessa et al., 2021). Furthermore, recent advances in the field of protein degradation pathways may help drive the targeting of these pathways in patients. Here we will discuss the role of protein degradation pathways associated with ER quality control: proteasomal ER-associated degradation (ERAD) and lysosomal ER-lysosomal associated degradation (ERLAD), how these pathways contribute to liver disease, and potential therapeutic strategies for targeting these pathways to limit disease progression.

### The ER is Critical for Hepatic Function

The ER plays several essential roles in the liver, thus maintaining its integrity is paramount for liver health. Hepatocytes contain large amounts of both rough ER and smooth ER, which serve different functions (Baiceanu et al., 2016; Schulze et al., 2019). The rough ER is marked by ribosomes, and is the main site of protein synthesis, folding, and secretion, while the smooth ER contains the majority of the machinery required for xenobiotic detoxification, including cytochrome p450 enzymes, as well as lipid synthesis (Schwarz and Blower, 2016). Both types of ER also play roles in cellular calcium homeostasis and stress-induced RNA degradation. The ER holds an extraordinary ability to expand when physiological or pathological stimuli require (Rutkowski and Hegde, 2010; Rutkowski and Kaufman, 2004). In the liver, specifically hepatocytes, the ER is essential for maintaining cellular function and integrity. Hepatocytes produce vast amounts of secreted proteins and lipoproteins which requires ER expansion and increased expression of chaperones proteins to assist with protein folding. The ER is also a critical site of protein quality control. Misfolded proteins are prevented from entering the secretory pathway and are instead targeted for degradation by the proteosome through ERAD, or the lysosome through ERLAD (Sun and Brodsky, 2019). ER quality control is crucial for cellular homeostasis, though the distinct contribution of ERAD or ERLAD to ER physiology or pathophysiology are unclear.

### ER Quality Control Pathways

#### ER-Associated Degradation (ERAD)

**FIGURE 1:**
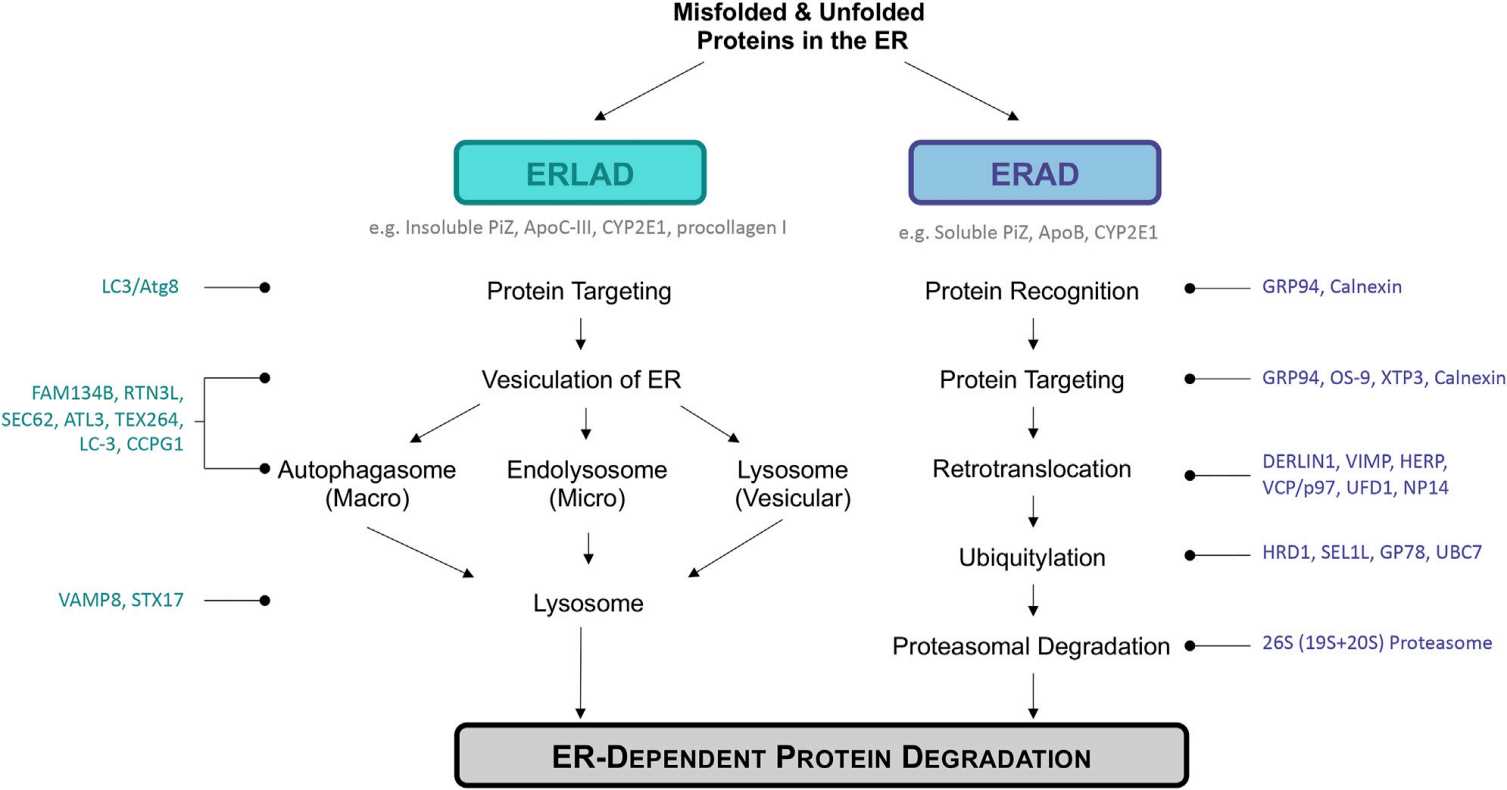
Overview of ER disposal pathways discussed: ERLAD and ERAD. ERLAD (ER lysosomal-associated degradation) and ERAD (ER associated degradation) pathways are the main degradation pathways of misfolded or aberrant proteins during chronic metabolic liver diseases. Examples of specific proteins degraded by each pathway are listed in dark grey under pathway headers, while molecules important during each step are listed to the left or right of the figure in either teal or dark blue. ERLAD can be broken into three sub-categories based on whether the process involves the autophagosomal (macro-ER-phagy), endolysosomal (micro-ER-phagy), or lysosomal (vesicular delivery) pathways. ERAD is composed of five different steps: protein recognition, targeting, retrotranslocation, ubiquitylation, and finally protein proteasomal degradation. All pathways work to degrade distressed proteins in order to return the cell to homeostasis and avoid cell death.

ERAD is a complex mechanism by which misfolded proteins in the ER are recognized, targeted, retrotranslocated to the cytoplasm, polyubiquitinated, and finally degraded by a proteasome (Figure 1). ERAD is also involved in regulating certain proteins based on metabolic signals, regardless of their configuration. In yeast, there are three types of ERAD: ERAD-L, ERAD-M, and ERAD-C, respectively targeting proteins with defects in their luminal region, membrane region, and cytoplasmic region for proteasomal degradation (Hwang and Qi, 2018). In mammals the distinction between ERAD-types is not as well established due to the complexity of ERAD mechanisms (Kwon et al., 2020) The ERAD machinery involves numerous chaperones and factors that help in the above processes in order to maintain cellular homeostasis and avoid triggering cellular death when the ER is overloaded with misfolded proteins (Baiceanu et al., 2016; Vembar and Brodsky, 2008; Olzmann et al., 2013; Fujii et al., 2018; Kuscuoglu et al., 2018; Fregno and Molinari, 2019; Ninagawa et al., 2021). ERAD canonically progresses through five stages: recognition, targeting, retrotranslocation/extraction, ubiquitinoylation, and degradation. These steps are extensively described elsewhere, thus we will briefly outline the process of ERAD here (Fregno and Molinari, 2019; Sun and Brodsky, 2019; Molinari, 2021).

Recognition is the act of identifying an ER located protein that is misfolded and needs to undergo degradation. Recognition is based on an abnormal conformation of mannose units by proteins such as OS-9 (OS9 endoplasmic reticulum lectin), or through prolonged cycling through chaperone proteins such as calnexin or calreticulin. Protein recognition is generally done by either heat shock proteins (HSP) such as HSP70 or HSP90 or chaperons such as calnexin, calreticulin, or BiP (immunoglobulin-binding protein). Targeting is the interaction of recognized proteins with the retrotranslocation machinery. In some cases the ERAD machinery combines recognition and targeting as the same complexes are used in both. In mammals, lectins such as OS-9, XTP3-B (endoplasmic reticulum lectin 1), and mannosidases such as ERManI (ER α-1,2-mannosidase 1) and EDEM1-3 (ER degradation-enhancing α-mannosidase-like) have been implicated in protein targeting. These factors recognize the number and organization of mannose moieties on the misfolded protein and based on this information target it for degradation. Retrotranslocation/Extraction is the act of moving the targeted protein from the ER lumen or ER membrane to the cytoplasm. It is essential that the protein targeted for degradation be moved to the cytoplasm as ubiquitination ligase complexes exists only there, as does the proteasome. Proteins involved in retrotranslocation/extraction in mammals are: VIMP (VCP-interacting membrane protein), Derlin 1-3, VCP (valosin-containing protein), Sec61 complex, and HRD1. Proteins undergo Ubiquitinoylation once retrotranslocated into the cytoplasm, which marks them for proteasomal degradation. In mammals E1-E4 ubiquitin enzymes are involved, such as the conserved E3 ubiquitin ligase complex HRD1-SEL1L. Finally proteins that have been ubiquitinylated undergo proteasomal degradation by the 26S proteasome a complex formed by two 19S subunits and one central 20S unit (Baiceanu et al., 2016; Vembar and Brodsky, 2008; Olzmann et al., 2013; Kuscuoglu et al., 2018; Ninagawa et al., 2021). Together these five stages—recognition, targeting, retrotranslocation/extraction, ubiquitinoylation, and degradation—of ERAD allow for proteins to be swiftly targeted for proteasomal degradation whether located in the ER lumen or ER membrane allowing ER homeostasis to reestablish preventing liver injury.

#### ER-to-Lysosomal Associated Degradative (ERLAD)

ER-to-Lysosomal-Associated Degradative (ERLAD) pathways describe a subset of processes that involve targeting of proteins in the ER lumen/membrane or the ER membrane itself for lysosomal degradation (Figure 1). These can involve engulfment of the ER by autophagosomes followed by lysosomal degradation (macro-ER-phagy), direct targeting of ER membrane to the lysosomes (micro-ER-phagy), and LC3/Atg8-dependent vesicular deliver. These processes have been implicated in several non-physiological states, such as in the presence of mutated/misfolded proteins *in vitro*, as well as with ER turnover following ER expansion in response to stress or increased secretion. While macro-ER-phagy is the most widely studied of the ERLAD pathways, each of the ERLAD pathways have been implicated in liver disease.

Macro-ER-phagy describes a set of processes that involve targeting of ER membrane or ER-associated proteins for autophagic engulfment followed by lysosomal degradation (Fregno and Molinari, 2018; Fregno and Molinari, 2019; Sun and Brodsky, 2019). These processes have been implicated in several physiological and pathological states, such as in the presence of mutated/misfolded proteins *in vitro*, as well as with ER turnover following ER expansion in response to stress or increased secretion (Sun and Brodsky, 2019; Fregno and Molinari, 2019; Fregno and Molinari, 2018).

In macro-ER-phagy, autophagic membranes are recruited directly to the ER by membrane-localized receptors, which typically bind to LC-3/Atg8 on autophagic membranes through LC-3 interacting regions (Sun and Brodsky, 2019; Grumati et al., 2018). Several membrane-localized ER-phagy receptors have been identified, including FAM134B/RETREG1 (Family With Sequence Similarity 134, Member B/Reticulophagy Regulator 1), CCPG1 (Cell Cycle Progression 1), TEX264 (Testis Expressed 264), SEC62, ATL3 (Atlastin 3), and the long isoform of RTN3 (Reticulon 3L) (Khaminets et al., 2015; Fumagalli et al., 2016; Grumati et al., 2017; Grumati et al., 2018; Smith et al., 2018; An et al., 2019; Chen et al., 2019; Chino et al., 2019). These receptors accumulate, leading to membrane deformation. The subsequent targeting of the ER for degradation often occurs through recruitment of ATG8/LC-3 proteins located on autophagic membranes, followed by engulfment and trafficking of ER membrane to the lysosome (Sun and Brodsky, 2019; Grumati et al., 2018; Fumagalli et al., 2016; Fregno et al., 2018; Omari et al., 2018; Loi et al., 2019; Fregno et al., 2021). The mechanisms regulating engulfment/scission are unknown. Several membrane-localized ER-phagy receptors have luminal domains which may facilitate specific targeting of proteins for ER-phagy (CCPG1, FAM134B-2, TEX264. Other ER-phagy receptors do not contain a luminal domain, and instead use intermediaries to target cargo for degradation. This includes FAM134B targeting procollagen I for degradation through Calnexin (Forrester et al., 2019). In addition to cargo specificity, ER-phagy receptors show specificity based on ER morphology, with ATL3, CCPG1, and RTN3L acting at ER tubules, FAM134B facilitating ER-phagy along ER sheets, and TEX264 recruiting autophagosomes to three-way junctions of the ER (Grumati et al., 2017; Khaminets et al., 2015; Smith et al., 2018; An et al., 2019; Chen et al., 2019; Chino et al., 2019; Liang et al., 2018). This distinct localization may also have implications for specific cargo that is recruited for ER-phagic-degradation, or specific processes that require distinct ER morphology. Recently, cytoplasmic proteins that participate in ER-phagic degradation were identified (Sequestosome 1/p62, C53, CALCOCO1 (Calcium Binding And Coiled-Coil Domain 1), and CALCOCO2) as well as several potential ER-phagic proteins identified in a recent screen; however, the distinct mechanisms for how these non ER-membrane bound proteins drive ER-phagy is unclear (Yang et al., 2016; Liang et al., 2020; Nthiga et al., 2020; Stephani et al., 2020). Due to the importance of the ER for hepatocyte function, ER-phagy is critical to maintain a healthy liver; however, little is known regarding the role and regulation of this degradative process in hepatocytes or non-parenchymal liver cells.

Micro-ER-phagy and LC3/Atg8-dependent vesicular delivery described processes where the ER is directly targeted to the lysosome without autophagic engulfment (Fregno and Molinari, 2019). Micro-ER-phagy has been observed during recovery from ER stress, and in response to aggregation of misfolded proteins in the ER that are resistant to ERAD, such as in AATD or procollagen I which will be discussed later in this review (Fregno and Molinari, 2018; Fregno et al., 2021). In mammals, the micro-ER-phagy and LC3-Atg8-dependent vesicular delivery pathways currently identified involve LC-3 lipidation, which targets the ER to interact with endolysosomal membranes followed by fusion of the membranes and degradation of the target proteins (Molinari, 2021). This process was found to degrade protein aggregates through a non-rapamycin responsive mechanism, suggesting that the autophagosome is not involved (Fregno and Molinari, 2018; Fregno et al., 2021). FAM134B was found to play a role not only in macro-ER-phagy, but also LC3/Atg8-dependent vesicular delivery. It remains unclear whether this process occurs under physiological conditions, or whether increased aggregation of misfolded proteins in the ER is required, but its potential role in liver pathology warrants further investigation.

### Regulation of ER Quality Control Pathways by the Unfolded Protein Response

A major goal of ERAD and ERLAD is to remove misfolded proteins from the ER, thus it is unsurprising that these processes are regulated in part by the unfolded protein response (UPR). As the UPR is critical for liver physiology and is dysregulated in chronic liver disease, regulation of ER quality control pathways by the UPR is important to discuss. The UPR is initiated by ER stress, occurring through excess unfolded or misfolded proteins accumulating within the ER (Hetz et al., 2020; Schwarz and Blower, 2016). The UPR is propagated through three canonical ER membrane proteins: IRE1α (inositol requiring enzyme 1α), PERK (protein kinase RNA-like ER kinase), and ATF6α (activating transcription factor 6 alpha) (Schwarz and Blower, 2016; Hetz et al., 2020). Signaling through these pathways promote expression of chaperone proteins as well as proteins involved in degradative processes, while limiting non-essential protein translation. Furthermore, the UPR plays a critical role in liver disease progression and fibrosis, thus the role of the UPR in regulating protein degradation through ERAD and ERLAD is important to discuss (Maiers and Malhi, 2019). IRE1α and ATF6α are the primary regulators of ERAD in response to ER stress. Upon sensing ER stress, IRE1α splices XBP1 mRNA to induce translation of the active XBP1 transcription factor (Tirosh et al., 2006; Jurkin et al., 2014). XBP1 translocates into the nucleus and promotes transcription of several ERAD-associated genes, including ERdj3 (Endoplasmic Reticulum DnaJ Homolog 3), ERdj4, EDEM1, UBE2E1 (Ubiquitin Conjugating Enzyme E2 E1), and HERPUD1 (Homocysteine Inducible ER Protein With Ubiquitin Like Domain 1) (Oda et al., 2006; Yamamoto et al., 2007; Park et al., 2021). ATF6α is also activated in response to ER stress, promoting ATF6α trafficking to the Golgi. At the Golgi, ATF6α is cleaved and the active portion of ATF6α is released, subsequently entering the nucleus and promoting expression of XBP1 and several ERAD-associated genes (Yamamoto et al., 2007; Yoshida et al., 1998; Haze et al., 1999; Wang et al., 2000; Shen et al., 2002). Induction of the ERAD machinery is critical for identification, translocation, and degradation of misfolded proteins located within the ER lumen/membrane to relieve ER stress (Hwang and Qi, 2018). UPR regulation of ERAD likely directly contributes to hepatic steatosis and liver disease. Mice lacking hepatocyte expression of IRE1α exhibited worsened steatosis in response to ER stress (Zhang et al., 2011). Furthermore, proteosome inhibition was sufficient to promote lipid accumulation in the liver, which was exacerbated with IRE1α knockout (Zhang et al., 2011). Subsequent studies have investigated the role of IRE1α in steatosis; however, no reports have studied the link between IRE1α-regulation of ERAD to steatosis development (Maiers and Malhi, 2019).

Less is known regarding UPR regulation of ERLAD. ER stress is tightly linked to autophagy and lysosomal degradation, through UPR-mediated upregulation of LC-3B cleavage and expression of SQSTM1/p62 and Beclin (Kim and Kim, 2020; Xia et al., 2020). The ER-phagy receptor CCPG1 is upregulated by ER stress in mammalian cells potentially through XBP1, while FAM134B expression is upregulated by c/EBPβ, which is downstream of the UPR sensor ATF6α (Smith et al., 2018; Kohno et al., 2019). No other direct link between the UPR and expression of ER-phagy receptors is reported, though the UPR likely plays an important role in regulating ERLAD initiation and flux through ERLAD pathways as well.

### Physiological ERAD and ERLAD in the Liver

Under physiological settings, ERAD and ERLAD serve important roles in hepatocytes (Moore and Hollien, 2012). The hepatocyte is a professional secretory cell, producing an estimated 13 million secretory proteins each minute (Schulze et al., 2019). Both the smooth and rough ER expand to accommodate induced secretion of proteins, but also need degradative pathways to remove misfolded proteins, facilitate membrane turnover, and decrease ER size upon removal of the secretory stimuli (Sun and Brodsky, 2019; Moore and Hollien, 2012). ER stress is prevalent in physiological conditions, including the post-prandial state where the increased lipid and carbohydrate presence in hepatocytes further stress the ER (Deng et al., 2013). While the transcriptional programs activated by the UPR have been, and continue to be, extensively studied in the liver, the degradative pathways activated to remove misfolded or excess proteins are understudied in their relevance to liver physiology.

*ERAD*: Numerous *ERAD* components are conserved across mammals, and ubiquitously expressed across tissues. In particular, the E3 ubiquitin ligase HRD1 and its cofactor SEL1L are considered the most conserved ERAD system that facilitates retrotranslocation of misfolded proteins from the ER and subsequent proteasomal degradation (Vembar and Brodsky, 2008). In hepatocytes, HRD1/SEL1L regulates responses to fasting and feeding, regulating protein levels of the transcription factor CREBH (cyclic adenosine monophosphate (c-AMP)-responsive element binding protein H) which in turn increased transcription of FGF21 (Fibroblast growth factor 21) (Bhattacharya et al., 2018). In this manner, increased expression of HRD/SEL1L enhanced ERAD-mediated degradation of CREBH during feeding regulates metabolism in hepatocytes (Bhattacharya et al., 2018). ERAD is also associated with cholesterol biosynthesis, through targeting key enzymes involved in cholesterol synthesis, such as squalene epoxidase and 3-Hydroxy-3-Methylglutaryl-CoA Reductase for proteasomal degradation (Tan et al., 2019). Based on these physiological roles, inhibition, or suppression of ERAD could significantly influence metabolism and cholesterol synthesis, potentially driving liver disease.

*ERLAD:* Expression of the major ER-phagy receptors is relatively ubiquitous, with both an N-terminal truncated isoform of FAM134B (FAM134B-2) and CCPG1 showing some enrichment in the liver (Ma et al., 2018; Kohno et al., 2019). Little is known regarding the physiological or pathophysiological roles of ER-phagy receptors in liver disease, but two key studies recently provided insight into how ER-phagy regulates liver physiology. First, Kohno et al. found that FAM134B-2 increased in response to starvation in mouse livers, and this occurred through C/EBPβ-mediated transcription (Kohno et al., 2019). FAM134B is also associated with activation of the transcription factors transcription factor EB (TFEB) and Transcription Factor Binding To IGHM Enhancer 3 (TFE3) which are established regulators of autophagic genes in response to starvation (Kohno et al., 2019; Cinque et al., 2020). Supporting a physiological role for ER-phagy receptors in the liver, ER microsomes were isolated from FAM134KO or wild type mice after fasting. Proteomic analysis of proteins from the microsomes revealed 40 proteins enriched in FAM134B KO livers compared to wild type, including Apolipoprotein C-III (ApoC-III) which will be discussed later for its role in NAFLD pathogenesis (Kohno et al., 2019). Thus, FAM134B-2 may be activated under conditions of both ER stress and starvation/amino acid depletion.

## ER Quality Control Pathways and Chronic Liver Disease

ER stress and chronic liver disease are pathologically linked, with pathogenic stimuli leading to ER stress, and ER stress driving liver disease pathology. The mechanisms through which injurious stimuli drive chronic liver disease through ER stress are described in detail elsewhere, so we will only include a brief discussion, focusing more on the role of ER quality control pathways in this review (Baiceanu et al., 2016; Maiers and Malhi, 2019; Kim and Kim, 2020; Flessa et al., 2021). ER stress occurs in response to increased triglyceride and cholesterol accumulation in hepatocytes as well as chronic alcohol consumption. ER stress and UPR signaling further potentiate liver damage through promoting hepatocyte apoptosis downstream of ATF4/CHOP signaling, inducing release of pathogenic extracellular vesicles through IRE1/XBP1, regulating steatosis, activating inflammatory pathways, and promoting fibrogenesis (Ji et al., 2005; Oyadomari et al., 2008; Cazanave et al., 2010; Pfaffenbach et al., 2010; Cazanave et al., 2011; Li et al., 2011; Malhi et al., 2013; Xiao et al., 2013; Toriguchi et al., 2014; Kakazu et al., 2016; Rahman et al., 2016; Shan et al., 2017; Dasgupta et al., 2020; Duwaerts et al., 2021). ATF6α is involved in lipid synthesis regulation, with ATF6α loss in hepatocytes limiting steatosis, but may also promote fibrogenesis in response to liver injury (Rutkowski et al., 2008; Yamamoto et al., 2010; Chen et al., 2016a; Xue et al., 2021). contributing to NAFLD development and progression. Indeed, ER stress is linked with the progression of NAFLD to NASH, as well as fibrosis progression, thus understanding the contribution of these pathways to chronic liver disease, as well as targeting these pathways to limit progression and/or promote regression of liver disease is of paramount importance. A critical and understudied UPR regulated process are the ER quality control pathways. The studies described earlier implicate ERAD and ERLAD as critical regulators of liver physiology, and potential drivers of liver pathology. We will further discuss the established and potential pathological roles for ERAD and ERLAD in genetic and metabolic liver disease, including AATD, NAFLD/NASH, ALD, and fibrosis. Finally, we will discuss these processes as potential therapeutic targets for limiting progression of chronic liver disease.

## Alpha-1 Antitrypsin Deficiency

Alpha-1 antitrypsin (AAT) is a serine protease inhibitor important in degrading neutrophilic elastase in the lung. Under normal conditions it is synthetized and secreted by hepatocytes, but several mutations can exist in the gene that encodes AAT, *Serpina1* (Pi), which leads to diseases of both the lung and liver. The most severe form of AATD occurs with homozygous expression of ZZ alleles (protein: PI-Z) instead of the normal MM alleles, which leads to a Glu342Lys transformation. AATD affects 1/2,000-1/5,000 people world-wide (Manne and Kowdley, 2020). This missense mutation induces an accumulation of AAT PI-Z aggregates within the hepatocyte, specifically within the ER. Hepatic accumulation of AAT PI-Z leads not only to ER stress and subsequent liver disease but also to a deficiency in circulating and pulmonary AAT, which ultimately leads to lung disease (Teckman et al., 1996; Silverman and Sandhaus, 2009; Manne and Kowdley, 2020; Narayanan and Mistry, 2020; Patel et al., 2021). Progression of AATD can range from pediatric jaundice to the development of hepatocellular carcinoma in adulthood (Narayanan and Mistry, 2020). Hepatocytes attempt to control the accumulation of AAT aggregates *via* two main mechanisms—ERAD and macro-ER-phagy, though a direct lysosomal pathway has recently been implicated. For years ERAD was the favored mechanism through which an attempt at reestablishing protein homeostasis during AATD occurred. However, studies demonstrated that the preferred mechanism of AAT protein degradation mechanism is dependent on the state in which the PI-Z mutant proteins are found. When proteins remain soluble in monomeric or oligomeric form, they undergo ERAD rather than autophagy (Perlmutter, 2006). Most hepatic therapies for AATD have focused on increasing autophagy to reduce hepatocyte aggregates and the liver burden. The role of ERAD and ERLAD are best studied in AATD compared to other liver diseases, and may provide critical insight into how these pathways drive progression of other hepatic disorders. We will discuss these mechanisms, and lysosomal degradation, as they relate to AATD and liver disease.

### ERAD in AAT Deficiency and Liver Disease

Early accounts of ERAD during AATD demonstrated that PI-Z could be degraded through proteasomal degradation involving calnexin (Qu et al., 1996). Briefly, they described a phenomenon where PI-Z bound calnexin, which in turn promoted calnexin retrotranslocation and polyubiquitination. The ubiquitinated calnexin was then recognized by cytoplasmic proteasomes for degradation. From there the involvement of ERAD in the degradation of PI-Z during AATD only became more complex. Using an array of plasmids and HEK 293 cells, Shen et al. proposed that p97/VCP (valosin containing protein) were involved in the retrotranslocation of PI-Z, which was subsequently ubiquitinated by the E3 ubiquitin ligase GP78/AMFR (autocrine motility factor receptor) and UBE2G2 (ubiquitin-conjugating enzymes E2 7) (Shen et al., 2006). Further reports added to the ERAD machinery discovery. For example, several groups demonstrated that the E3 ubiquitin ligase HRD1/SYNV1 (synoviolin1) facilitated PI-Z degradation (Christianson et al., 2008; Iida et al., 2011; Wang et al., 2011). Christianson and colleagues, through a series of *in vitro* experiments, also described upstream events in the ERAD cascade for PI-Z degradation (Christianson et al., 2008). They established that the ER resident lectins OS-9 and XTP3-B, and ER resident chaperone glycoprotein GRP94/HSP90B1 (heat shock protein 90 beta family member 1) were responsible for delivering PI-Z to the SEL1L adaptor subunit of the SEL1L/HRD1 complex for ubiquitination and degradation. Derlin-1 has also been described as a part of the ERAD machinery involved in degrading PI-Z proteins (Lilley and Ploegh, 2005; Greenblatt et al., 2011). Finally a large complex of molecules termed, Complex I, was defined in ERAD of PI-Z (Ye et al., 2004; Lilley and Ploegh, 2005; Iida et al., 2011). Complex I was composed of: OS-9, SEL1L, HRD1 (which together form Complex II), in addition to HERP (Hes related family BHLH transcription factor with YRPW motif 2), Derlin-1, VIMP (selenoprotein S), p97/VCP, NPL4 (NPL4 homolog, ubiquitin recognition factor), UFD1 (ubiquitin recognition factor in ER associated degradation 1). Molecules involved in the ERAD of mutant AAT protein PI-Z during AATD are summarized. In summary ERAD plays a key role in degrading PI-Z soluble aggregates, helping alleviate hepatocellular ER stress during AATD.

### ERLAD in AAT Deficiency and Liver Disease

Accumulation of PI-Z leads to the formation of insoluble aggregates, which are resistant to ERAD (Perlmutter, 2006). Despite this insolubility, studies showed that hepatocytes shifted to promote degradation through ER-phagy (Teckman and Perlmutter, 2000). The Perlmuter group microscopically examined this degradative process in several different cell lines, PI-Z mutant mice, and liver samples from patients with AATD. They noted the formation of large insoluble aggregates of PI-Z that could be found within both the ER and autophagosomes. Around the same time, another group demonstrated that transfection of PI-Z into cells deficient for *Atg5* (autophagy related 5) led to increased PI-Z aggregate accumulation compared to WT cells (Kamimoto et al., 2006). In WT cells an increase in LC3^+^ autophagosomes, in the presence of PI-Z, was noted while *Atg5* deficient cell numbers increased indicating less autophagy occurring.

While not focused directly on macro-ER-phagy, additional studies have demonstrated that the autophagic regulator TFEB is actively involved in reducing PI-Z accumulation in hepatocytes (Pastore et al., 2012; Pastore et al., 2013). Both *in vitro* and *in vivo* studies showed that when *Tfeb* was transferred into cells or PI-Z mutant mice it significantly reduced PI-Z accumulation, and reduced liver disease and fibrosis *in vivo*. Finally, Feng et al. recently described the autophagic machinery involved in PI-Z degradation in more detail (Feng et al., 2017). Using a combination of HEK 293T and HepG2 cell lines, they established that SYVN1 was involved not only in ubiquitin tagging insoluble PI-Z for proteasomal degradation but also autophagic degradation. SYVN1 promoted the interaction of PI-Z with sequestosome1 (SQSTM1), which then interacted with LC3 at autophagosomes, leading to autophagic degradation of PI-Z. Further studies are needed to fully elucidate the exact autophagic and ER-phagic pathways involved in PI-Z degradation during AATD, but the groundwork has certainly been laid.

### Lysosomal Degradation Pathways in AAT Deficiency and Liver Disease

There exist very few accounts of the lysosomal pathway being involved in PI-Z degradation during AATD separate from autophagy, and of the accounts that exist, the degradation mechanisms differ. One group described an ERLAD (Fregno et al., 2018) mechanism, while the other maps out a Golgi-dependent degradation (Gelling et al., 2012) mechanism, both of which are discussed below.

ERLAD is the direct degradation of ER contained proteins, in this case polymeric PI-Z, by endolysosomes without the intervention of autophagosomes. Fregno et al. described the phenomena using a complex *in vitro* experimental system where they demonstrate that even in the absence of the autophagosome machinery PI-Z still undergoes lysosomal degradation. The paper establishes that single-membrane ER vesicles expressing FAM134B fusion with RAB7/LAMP1 expressing endolysosomes through the formation of a complex between FAM134B and LC3-II on the endolysosomes. The process is complete when the ER vesicle fully fuses to the endolysosomes through SNARE/STX17 and SNARE/VAMP8 localized on the ER vesicle and the endolysosomes, respectively (Fregno et al., 2018).

Gelling et al., also described PI-Z removal through lysosomal degradation, but through a mechanism that involves the Golgi. In a PI-Z yeast mutation system, the group screened mutations that disrupted PI-Z degradation, and found one target of interest—*Vps10* (Sortilin in humans). Sortilin is a protein involved in transporting misfolded proteins from the Golgi to vacuoles for secretion or degradation, it is localized in the late compartment of the Golgi. When they further investigated the role of sortilin in a rat hepatoma (RH-7777) cell line, they found that in a sortilin mutant PI-Z trafficking to the lysosome was disrupted, a defect that was corrected with sortilin overexpression. The group thus concluded that the Golgi trafficked PI-Z to lysosomes for degradation in a sortilin-dependent fashion (Gelling et al., 2012).

In conclusion, AATD burdens both the liver and lungs through the aggregation of AAT and lack of AAT circulation, respectively. Numerous groups have described that both ERAD and ERLAD are highly involved in reducing the liver burden during AAT aggregate accumulation within the ER of hepatocytes. ERAD being favored when aggregates remain small and soluble while ERLAD is used when aggregates are large and insoluble. Several animal models have tested drugs to enhance autophagy/macro-ER-phagy with promising results, while carbamazepine (CZB) is currently undergoing clinical trials for the treatment of liver disease during AATD.

## Non-Alcoholic Fatty Liver Disease

Non-alcoholic fatty liver disease (NAFLD) is an overarching term that incorporates both non-alcoholic fatty liver (NAFL) and non-alcoholic steatohepatitis (NASH). NAFL is the accumulation of fat, or steatosis, within the liver accompanied by little to no inflammation, while NASH is steatosis accompanied by inflammation, hepatocyte ballooning and death, as well as varying degrees of fibrosis (Kleiner et al., 2005). NASH is generally accompanied with other metabolic diseases such as obesity, diabetes mellitus, hyperlipidemia, and hypertension, and has become known as the hepatic manifestation of metabolic syndrome and insulin resistance (Kleiner et al., 2005). NAFL is generally thought of as a benign condition, while NASH is a substantial disease which can culminate into cirrhosis and hepatocellular carcinoma. As of today there are still no long-term treatment options for NASH at the exception of liver transplantation, though there is a considerable amount of research being done in the field to find treatment options. NAFLD is estimated to affect 24% of the world’s population, although this is likely an underestimation given that the disease is rarely accompanied by symptoms until it reaches the late stages (Younossi et al., 2016; Younossi et al., 2018). Of the 24% with NAFLD, 59% are estimated to have NASH. Furthermore, NAFLD disproportionately affects certain races, such as people of Hispanic descent, due to the strong genetic component of this disease (Younossi et al., 2016; Younossi et al., 2018). NASH is a complex disease which affects all aspects of the hepatocyte’s ability to respond to stress such as the overaccumulation of lipids. For example, in the case of excess lipids, the ER undergoes expansion. Expansion helps to accommodate for the increased physical lipid load and package and shuttle the lipid out of the hepatocyte; however, this expansion and increased load in turn triggers ER stress. ER stress plays a key role in NAFLD pathogenesis, in fact all arms of the UPR have been linked to NAFLD development, progression, and even fibrogenesis during NASH (Yamamoto et al., 2007; Zhang et al., 2011; Oyadomari et al., 2008; Rutkowski et al., 2008; Cazanave et al., 2010; Pfaffenbach et al., 2010; Yamamoto et al., 2010; Cazanave et al., 2011; Li et al., 2011; Malhi et al., 2013; Xiao et al., 2013; Toriguchi et al., 2014; Chen et al., 2016a; Kakazu et al., 2016; Rahman et al., 2016; Shan et al., 2017; Dasgupta et al., 2020; Duwaerts et al., 2021; Wang et al., 2018). In this section we will review the roles ERAD, autophagy, and lysosomal degradation pathways play in NAFLD pathogenesis, with an emphasis on lipid homeostasis.

### ERAD During NAFLD

Studies investigating the role of ERAD in NAFLD pathology have primarily focused around how ERAD regulates lipid homeostasis. Some of the earlier reports on ERAD during NAFLD focused on Apolipoprotein B (ApoB) (Fisher et al., 1997; Gusarova et al., 2001; Hrizo et al., 2007; Brodsky and Fisher, 2008). ApoB is the principal apolipoprotein in VLDL (very-low-density lipoprotein) and LDL (low-density lipoprotein) and is responsible for shuttling these lipoproteins through the secretory pathway. ApoB dysfunction is strongly associated with NAFLD development, with mutations in ApoB implicated as a genetic driver of NAFLD. It is important to note that enhanced ApoB degradation leads to increased accumulation of lipids within the liver as proper lipid secretion is impaired. Fisher and colleagues demonstrated that Hsp70, an ERAD chaperone, was essential for ApoB degradation in a HepG2 overexpression system (Fisher et al., 1997). A second ERAD chaperone, HSP90, was also implicated in ApoB degradation, with transfection of HSP90 into a rat hepatoma line, RH-7777 significantly increasing ApoB degradation (Gusarova et al., 2001). Inversely, chemically disrupting the interaction between ApoB and HSP90 with Geldanamycin significantly decreased ApoB degradation. Finally the group demonstrated in HSP90 and HSP70 mutant yeast cells that ApoB was not degraded, tying in with Fisher’s earlier work. Hrizo and group demonstrated that HSP110 played an essential role in stabilizing ApoB and decreased its degradation. They showed this in the cell line RH-7777 where they over expressed HSP110, which increased ApoB secretion (Hrizo et al., 2007). More recent work further tied ApoB degradation to NAFLD pathogenesis (Yamamoto et al., 2010). In an *Atf6* knock-out mouse model where ER stress was induced with tunicamycin (1 mg/kg) for one week, an increase in steatosis and liver injury occurred. Yamamoto and colleagues went on to demonstrate that this increase in steatosis was due to increased triglyceride and cholesterol accumulation, decreased β-oxidation, and decreased VLDL secretion due to ApoB destabilization. This group speculated that the observed pathogenesis was due to the role ATF6 plays in the transcription of ERAD associated genes, with ATF6α loss limiting the levels of ERAD machinery, subsequently destabilizing ApoB and decreasing lipid export from hepatocytes (Yamamoto et al., 2007). Other studies demonstrated that ERAD is involved in increased lipogenesis *via* INSIG1 degradation and subsequent SREBP-1c activation, or through reduced TG synthesis by destabilizing DGAT2 (diacylglycerol O-acyltransferase 2) which is responsible for the last step of TG synthesis, thus leading to fatty acid accumulation (Liu et al., 2012; Choi et al., 2014). Together these studies demonstrate that targeting ApoB to increase stabilization through the potential inhibition of ERAD during NAFLD may help alleviate the ER burden and overall cellular stress helping reduce cell death and injury.

A second ERAD-associated mechanism regulating lipogenesis involves the E3 ubiquitin ligase HRD1, however the role of HRD1 in NAFLD pathogenesis remains unclear due to conflicting reports (Wei et al., 2018; Li et al., 2021). Wei and colleagues published that liver-specific depletion of Hrd1 was beneficial to steatosis and insulin resistance (Wei et al., 2018). Liver-specific Hrd1 knock out mice fed a high fat diet displayed a significant decrease in steatosis, blood glucose levels, and expression of *de novo lipogenesis* genes compared to wild-type mice on the same diet. They proposed that Hrd1 is an important metabolic regulator, whose loss promotes AMPK and AKT hyperactivation, leading to increased lipogenesis. In contrast Li and colleagues demonstrate that in a genetic model of obesity and diabetes (db/db mice) Hrd1 is significantly decreased compared to wild-type mice (Li et al., 2021). They also establish that Hrd1 is essential for ubiquitin degradation of Acyl, which is important in *de novo lipogenesis.* In the natural absence of Hrd1, db/db mice displayed an increase in circulating Acyl which leads to increased lipogenesis. They further demonstrate their findings by overexpressing Hrd1 in db/db mice and showing a fully reversed phenotype. Very recently Yang and group established that the E3 ligase RNF5 was important in HRD1 ubiquitination and degradation (Yang et al., 2021a). Mice with liver specific *Rnf5* depletion developed NASH (increased steatosis, inflammation, and fibrosis) when fed a high fat-high cholesterol diet. Investigating human NAFLD samples, they measured significantly less RNF5 mRNA and protein in NASH than NAFL samples. This new data could point towards HRD1 having a detrimental role in NASH as indicated by Ye et al. While ERAD appeared detrimental to ApoB stabilization, its overall role in NAFLD pathogenesis may not be as clean cut. Given our current understanding of Hrd1 in NAFLD progression, it might be beneficial to increase ERAD rather than limit it as studies studying ApoB indicate. Thus targeting ERAD may not be an easy task in NASH.

Another protein of the ERAD machinery that has been directly linked to NAFLD pathogenesis and hepatocellular carcinoma (HCC) development is the E3 ubiquitin ligase GP78 (Zhang et al., 2015). Zhang and researchers found that 1 year old *Gp78* knock-out mice naturally developed obesity. The obesity was accompanied by steatosis, liver inflammation, fibrosis, and HCC. Indeed, they found that *Gp78* levels in human HCC inversely correlated with grade of HCC. They hypothesized that loss of *Gp78* decreased ERAD which induced chronic ER stress leading to NASH and HCC. Finally when investigating RNA sequencing datasets from studies that investigated NAFL and NASH patients comparing them to healthy controls or obese controls several genes involved in ERAD emerged in most studies searched. For example the following genes were often found upregulated: *Fbxo1*, *Ubxn1*, *Tmub2*, *Man1b1*, *Stub1* while these were found to be downregulated: *Derl2*, *Man1a2*, *Ube2g2*, *and Insig* (Table 1) (Baker et al., 2010; Liu et al., 2011; Starmann et al., 2012; Ahrens et al., 2013; Murphy et al., 2013; Arendt et al., 2015). For example *Stub1*/Chip plays an essential role in NASH pathogenesis. When *Chip* was knocked out in mice, they developed significantly more oxidative stress, steatosis, cell death, and fibrosis than their wild-type counterparts in the absence of other stimuli (Kim et al., 2016). As for *Ube2g2*, it is involved in cholesterol synthesis, specifically it degrades the rate limiting enzymes involved in this process (Tan et al., 2019; Miao et al., 2010) Future studies into the pathological roles of these differentially regulated ERAD proteins may reveal novel mechanisms of NAFLD progression and potential therapeutic targets for future studies and therapies.

**TABLE 1:** RNAseq Analyses of patients with NAFLD/NASH.

Dataset	Comparison	ERAD: Upregulated genes	ERAD: Downregulated genes	ER-phagy: Upregulated genes	ER-phagy: Downregulated genes
GSE 17470	NASH vs. Control	UBXN1, DNAJC10	DERL2, UBE2G2, SEL11, RNF103, INSIG1	—	—
GSE 24807	NASH vs. Control	UBXN1, DNAJC10, UBE2K, TMUB2	DERL2, UBE2G2, MAN1B1, DERL3, RNF170, SEL1L, RNF103, INSIG1	—	—
GSE 48452	NASH vs. Obese	FBXO2	MAN1A2, MAN1A1	—	—
	NASH vs. Steatosis	FBXO2, DNAJB14	—	ATL3, RTN3	CDK5RAP3/C53, CALCOCO1
	Steatosis vs. Obese	—	MAN1A2, RNF170	—	SEC62
GSE 89632	NASH vs. Healthy	FBXO2, AMFR, STUB1, TMUB2, RNF170, UBXN6, TRIM25, DERL3, DNAJB12, DNAJB14, TMEM129, DERL2, EDEM2, TRIM21, MAN1A2, RNF185	FAF2, UBE2J1, UBE2G2, MANC1, INSIG1, TRIM13	CCPG1, ATL2	RTN3, RETREG1
	Steatosis vs. Healthy	FBXO2, STUB1, RNF5, TRIM13, UBXN6, TMEM129, TMUB2, RCN3, DNAJB12, RNF170, RNF139	RHBDD1, UBE2G2, MANC1, INSIG	DDRGK1, TEX264	RTN3, RETREG1
	NASH vs. Steatosis	N/A	N/A	N/A	N/A
GSE 33814	NASH vs. Healthy	WFS1, FBXO2, MAN1B1, TMUB2, NPLOC4, MAN2B1, RCN3, SORT1	TRIM13, DNAJB14, RNF170, UBE2K, XBP1, MARCHF6, MAN1C1, INSIG1	ATL3, RTN3, CCPG1, CDK5RAP3/C53	RETREG1, SEC62, CALCOCO2
	NASH vs. Steatosis	FBOX1, RCN3, MAN1B1, TMUB2, MAN2B1,UBXN1, STUB1, DNAJC18, SORT1	ERLIN1, SYVN1, UBXN4, SEL1L, RNF103, HSPA5, MAN1C1, RNF170, UBE2K, RNFT1, DNAJB14, INSIG1	—	ATL2, SEC62, CALCOCO2
	Steatosis vs. Healthy	NPLOC4, MAN1B1, TRIM25, DERL2, MAN2C1, RNF185, RHBDD1, MAN2B1, TMUB2, DNAJB12, STUB1, DNAJB14, UBE2J2	RNF139, JKAMP, UBE2K, MAN1A1, UBXN8, MAN1C1, XBP1, MARCHF6	RTN3, CCPG1, CDK5RAP3/C53	DDRGK1, RETREG1, SEC62, CALCOCO2
GSE 49541	NAFLD with Advanced Fibrosis (Stage 3/4) vs NAFLD with Mild Fibrosis (Stage 1/2)	DNAJC10	MAN1C1	—	—
GSE 159676	NASH vs. Healthy	PRKN, DNAJB14, DERL3	BAG6, UBXN8, MAN1A1, UBXN4m TMEM129, ERLIN1, UBXN6, STUB1, DNAJB11, MAN1C1, EDEM2, OS9, INSIG1, UBE2D1	RTN3, ATL3	TEX264, SEC62, RETREG1, CALCOCO1, CALCOCO2, CDK5RAP3/C53

### Autophagy/Macro-ER-Phagy During NAFLD

The role of autophagy in NAFLD progression has been an exciting area of research in the past decade, though the contribution of ER-phagy to NAFLD and NASH development is unclear. One of the first reports of autophagy in the liver during NAFLD was by Singh and colleagues in 2009 (Singh et al., 2009). Since then hundreds of papers have been written on the subject, of which several reviews of note (Flessa et al., 2021; Wu et al., 2018; Ramos et al., 2021). In this seminal paper, Singh et al. demonstrated in both *in vitro* and *in vivo* settings that the inhibition of autophagy led to increased lipid accumulation, reduced β-oxidation, and reduced VLDL secretion in hepatocytes coining the term “macrolipophagy”. For example, culturing primary hepatocytes from *Atg5* knock out mice in the presence of oleate led to significant accumulation of triglycerides compared to wild-type hepatocytes. The group went on to demonstrate that autophagy was required for β-oxidation of lipids by adding siRNA for *Atg5* to cultured hepatocytes in the presence of MCD (methionine and choline deficient) media. They also showed microscopically that lipid droplets were associated with autophagic markers such as LC3 and LAMP1. Finally in mice fed high fat diets (HFD), autophagy decreased in the presence of HFD, leading to increased lipid droplet accumulation, which further inhibited autophagy, highlighting a vicious cycle (Singh et al., 2009). Soon after, a report from Liu and colleagues demonstrated the integral role insulin plays in hepatic autophagy (Liu et al., 2009). Autophagy was diminished in mice fed a HFD to induce insulin resistance, but when insulin was inhibited chemically with streptozotocin, autophagy was restored (Liu et al., 2009). Yang et al. further demonstrated the importance of insulin in hepatic autophagy, revealing that *in vivo* loss of *Atg7* increased both ER stress and insulin resistance (Yang et al., 2010). They also showed that all markers of autophagy were dysregulated demonstrated in an ob/ob model, resulting in overall downregulated autophagy.

Numerous other reports employing high fat or methionine-choline deficient dietary models in mice or rats have come to the same conclusions as Singh and colleagues, in the presence of a NAFLD stimulus less autophagy is observed, and consequent lipid accumulation is noted (Koga et al., 2010; Wang et al., 2010; Gonzalez-Rodriguez et al., 2014; Simon et al., 2014). Through these reports the autophagic machinery in NAFLD has been well documented and includes classic autophagic markers such as ATG5, ATG7, BECLIN-1, p62/SQSTM1, LC3-II/I, and S6K1(Singh et al., 2009; Yang et al., 2010; Gonzalez-Rodriguez et al., 2014). Finally Gonzalez-Rodriguez et al. studied liver samples from healthy control, NAFL, and NASH patients and established that patients with NASH had significantly less autophagy than both other groups as seen by their increased p62 protein levels, increased phopspho-mTOR, increased phospho-S6K1, and decreased mRNA levels of Beclin-1 (Gonzalez-Rodriguez et al., 2014).

While not as robustly studied, macro-ER-phagy may indeed play a crucial role in NAFLD pathogenesis. As ApoB is highly regulated by ERAD, another apolipoprotein is regulated by macro-ER-phagy. ApoC-III is involved in lipid homeostasis through two distinct functions: (Asrani et al., 2019) it inhibits hepatic lipase, and (Maiers and Malhi, 2019) inhibits uptake of triglyceride-rich particles. If ApoC-III is not tightly regulated it can lead to hypertriglyceridemia and an increased risk of cardiovascular disease (Boren et al., 2020). Interestingly, a polymorphism has been found in ApoC-III lipoprotein which predisposes to NAFLD (Petersen et al., 2010). Recently published work has pointed to a role for macro-ER-phagy in the regulation of lipoprotein ApoC-III (Kohno et al., 2019). Specifically the group describes that ApoC-III is degraded in a FAM134B-2-dependent manner. This means macro-ER-phagy plays a key role in regulating lipogenesis in hepatocytes, lending it a potential central role in NAFLD pathogenesis. Changes in macro-ER-phagy during NAFLD progression could further lead to the progression of NAFLD through, for example, the regulation of ApoC-III levels in hepatocytes. A role for ERLAD in NAFLD/NASH pathogenesis is further supported by RNA sequencing data from groups that compared NASH to healthy controls. Numerous ER-phagy receptors were differentially regulated, such as ATL3, SEC62, and RTN3 (Table 1) (Baker et al., 2010; Liu et al., 2011; Starmann et al., 2012; Ahrens et al., 2013; Murphy et al., 2013; Arendt et al., 2015). Again, this points towards macro-ER-phagy playing an essential role during NASH and that targeting the machinery to treat NASH is a highly understudied area.

### Lysosomal Degradation During NAFLD

The role of lysosomal degradation is less studied in NAFLD pathogenesis, with even less known regarding the role of ERLAD. Despite being understudied, research shows that lysosomal degradation plays a principal role in NAFLD pathogenesis. Key proteins of interest in lysosomal degradation are RABs (Ras-related protein Rab), Sortilin (VSP10), and SIMPLE (small integral membrane protein of lysosome/late endosome) (Strong et al., 2012; Zeigerer et al., 2012; Du et al., 2020; Song et al., 2021; Vos and van de Sluis, 2021). RABs are a large family of GTPase essential in early endosome, late endosomes, and lysosome formation. They are located on the surface of endosomes/lysosomes and are essential in connecting with lipid droplets and their trafficking and degradation (Liu et al., 2007). Such is the case of *Rab5*, which plays a role in the actual number of endosome/lysosomes present in hepatocytes (Zeigerer et al., 2012). In fact Zeigerer et al. found a loss of the endosomal pathway in the absence of Rab5. They also demonstrate that in the absence of *Rab5*, LDL was not able to undergo endocytosis into the primary hepatocytes used for the experiment, leading to an increased amount of circulating LDL.

Strong and colleagues investigated the role of Sortilin in lysosomal degradation of lipids (Strong et al., 2012). Sortilin is a lysosomal sorting protein found in both the Golgi and plasma membranes and is important in trafficking to the lysosome. Interestingly, this group demonstrated that increased Sortilin expression by AAV transfection into wild-type mice lead to decreased ApoB secretion and increased LDL catabolism, both were due to increased lysosomal degradation. A natural occurring mutation in Sortilin in humans significantly reduces coronary heart disease, taken together this pathway may be an interesting target for NAFLD therapeutics. Another group investigated the role SIMPLE played in NAFLD (Song et al., 2021). Song et al. investigated levels of SIMPLE in normal and NASH patient samples and found, as in their NAFLD mouse models, a significant decrease in the protein in diseased individuals. They further demonstrated that in a SIMPLE knock-out mouse model fed either an MCD, HFD, or HFHC diet they developed significant steatosis, insulin resistance, inflammation, and fibrosis. They went on to demonstrate that SIMPLE interacts with EGFR (epidermal growth factor receptor) and regulates its degradation. In the absence of SIMPLE EGFR was found hyperactivated which led to NAFLD.

When taken together it is evident that all three pathways for degradation of ER membrane/cargo are heavily involved in NAFLD pathogenesis, especially in the role they play in lipid homeostasis.

## Alcoholic Liver Disease

ALD develops in response to chronic, excessive alcohol consumption, the prevalence of which is rising rapidly (Nagy et al., 2016). Both acute and chronic ethanol consumption lead to ER stress, and in turn, resulting UPR signaling drives ALD pathogenesis (Barak et al., 1996; Werstuck et al., 2001; Ji and Kaplowitz, 2003; Ji et al., 2011; Fernandez et al., 2013; Tsedensodnom et al., 2013; Howarth et al., 2014; Li et al., 2016; Masouminia et al., 2016; French et al., 2017; Song et al., 2020). Furthermore, ethanol-induced hepatocyte death occurs through UPR-upregulation of CHOP (Ji et al., 2005). Liver damage in ALD primarily occurs through ethanol metabolism which releases reactive oxygen species (ROS). The majority of ethanol metabolism occurs through alcohol dehydrogenase (ADH) and aldehyde dehydrogenase (ALDH) 1 and 2, but in conditions of excess ethanol, cytochrome p450 (CYP) enzymes, particularly CYP2E1, metabolize ethanol and produce ROS. Increased ROS in hepatocytes leads to mitochondrial damage, interference with protein folding, ER stress, and hepatocyte death, which in turn drives ALD progression (Wu and Cederbaum, 1996; Nagy et al., 2016; Doody et al., 2017; Lu and Cederbaum, 2018). Other ethanol-induced drivers of ER stress are protein accumulation and adduct formation, both of which impair hepatocyte function and lead to hepatocyte apoptosis, which may be aggravated by impaired ER quality control (Chen et al., 2016b; Masouminia et al., 2016; French et al., 2017).

Without dismissing other ethanol metabolizing pathways, much of our focus will be on CYP2E1 due to its relationship with ERAD and autophagy (Kwon et al., 2020; Correia et al., 2014). CYP2E1 plays a modest role in ethanol oxidation under normal conditions; however, this role increases as alcohol exposure increases. Ethanol increases CYP2E1 production, and in turn, increased CYP2E1 levels or activity drives hepatocyte injury. Conversely, deletion or inhibition of CYP2E1 limits hepatocyte death and alcohol-induced injury (Lu and Cederbaum, 2018; Song et al., 2019). The deleterious effect of increased CYP2E1 on hepatocyte viability suggests that drugs aimed at mechanisms regulating CYP2E1 protein stability could improve the disease phenotype.

### ERAD and ALD

CYP2E1 is localized at the ER membrane where it metabolizes ethanol and other xenobiotics. As an ER membrane protein, CYP2E1 undergoes turnover through ERAD under physiological conditions, but this turnover is increased with CYP2E1 damage or inactivation (Kwon et al., 2020; Correia et al., 2014). CYP2E1 metabolism of ethanol and subsequent generation of ROS damages CYP2E1, leading to degradation by ERAD (Kwon et al., 2020; Correia et al., 2014). CYP2E1 is targeted for ERAD through an initial phosphorylation event mediated by PKA and PKC, followed by ubiquitination by E2/E3 complexes (Kwon et al., 2019). These complexes include p97/Ufd1/Npl4-AAA ATPase, UbcH5a/Hsp70/CHIP, and UBC7/gp78/AMFR. Ubiquitination is followed by extraction of CYP2E1 from the ER membrane and subsequent proteasomal degradation. Loss of any of these complexes increases CYP2E1 stability, which in turn worsens drug-induced liver damage in murine models (Kwon et al., 2019; Ballinger et al., 1999; Connell et al., 2001; Jiang et al., 2001; Murata et al., 2001; Morishima et al., 2005; Kim et al., 2010). Analyses of RNAseq from patients with alcoholic hepatitis showed increased expression of numerous ERAD genes, including those involved in CYP2E1 turnover (*AMFR* and *VCP*), though this reversed in patients with alcoholic cirrhosis, who displayed reduced expression of *Stub1*/CHIP (Table 2) (Bourd-Boittin et al., 2011; Caillot et al., 2009a; Trepo et al., 2018; Hyun et al., 2020; Affo et al., 2013). Increased expression of ERAD genes that target CYP2E1 for degradation may reflect an adaptive mechanism of hepatocytes to remove excess CYP2E1 induced by alcohol exposure. The shift in ERAD gene expression in cirrhotic patients mirrors what we observed in other cirrhotic patients (Table 3), suggesting that regulation of ERAD changes with disease severity (Caillot et al., 2009a; Bourd-Boittin et al., 2011; Affo et al., 2013; Trepo et al., 2018; Hyun et al., 2020). The mechanism behind this shift is unclear but could correspond with a shift away from cells trying to adapt and resolve cellular damage, to other, more pathological signaling pathways. Other key observations from our RNAseq analyses were dysregulated expression of several mannosidases (*MAN1A1*, *MAN1A2*, *MAN2A2*, *MAN1B1*, *MAN2C1*) and ubiquitin ligases (*UBE2N*, *UBXN8*, *UBE2J1*, *UBE2J2*). Increased expression of the ERAD machinery could also be downstream of ER stress and activation of the UPR in response to hepatocyte injury. How the ERAD machinery is regulated in response to acute and chronic alcohol exposure, as well as in early and late stages of alcoholic liver disease, would provide additional insight into the role of this process in disease progression.

**TABLE 2:** RNAseq analyses from patients with Alcohol-related liver disease.

Dataset	Comparison	ERAD: Upregulated genes	ERAD: Downregulated genes	ER-phagy: Upregulated genes	ER-phagy: Downregulated genes
GSE 28619	Alcoholic hepatitis vs. Control	TRIM13, DNAJC10, TRIM25, UBE2N, UBXN8, FBXO2, JKAMP, DNAJB11, TRIM21, AMFR, UFD1, DERL2, HSPA8, RHBDD1, DNAJB14, VCP, EDEM3, SEL1L, HSPA5, NPLOC4, ERLEC1, FAF2, UBE2J1, UBE2D1	UBXN6, HERPUD1, MARCHF6, ENF5, UBE2K, MAN1A1, MAN2C1, MAN2A2, MAN1C1, INSIG1	ATL3	RETREG1, ATL2
GSE 143318	Alcoholic hepatitis vs. Control	AMFR, EDEM1, MAN1A1, TRIM25, MAN1A2, RNF5, VCP, DNAJB11, STT3B, NPLOC4, EDEM3	—	DDRGK1	—
GSE 103580	Alcoholic steatosis vs. mild alcoholic hepatitis	SEL1L, TRIM13	TMEM67, DNAJC10	CCPG1	—
	Alcoholic cirrhosis vs. alcoholic steatosis	DNAJC10, EDEM3, MAN1B1, TMEM67, DNAJC18, ERLEC1, RNFT1, UBE2J2, UBE2J1	STUB1, UBXN6, UBE2K	—	CCPG1, SEC62
	Alcoholic cirrhosis vs. mild hepatitis	INSIG1, TRIM13, DNAJC10, RNFT1, UBE2G2, SEL1L, MAN2C1, EDEM3, EDEM1, RHBDD1, DNAJC18, STT3B, ERLEC1, UBE2J1, RCN3	STUB1, DNAJB12, UBXN6, SYVN1	—	—
GSE 10356	Alcoholic cirrhosis vs. normal	MAN1A2, HSPA5, EDEM1	TRIM21	—	—
GSE 24667	Alcoholic cirrhosis vs. normal	MAN2C1, MARCHF6, PRKN, FBXO2, UBE2N, WFS1	TRIM21, TRIM13, SEL1L	—	—

**TABLE 3:** RNAseq Analyses of Cirrhotic livers or Hepatic Stellate Cells.

Analysis of whole liver						
Dataset	Comparison	ERAD: Upregulated genes	ERAD: Downregulated genes		ER-phagy: Upregulated genes	ER-phagy: Downregulated genes
GSE 14323	Cirrhotic vs. Healthy	RCN3, UBE2N, UBE2D1	VCP, MAN2A2, UBE2J1, EDEM2, XBP1, WFS1, UBXN4, NPLOC4, EDEM3, DNAJC10, DERL2, MAN1A2, HSPA5, EDEM1, MAN1A1, AMFR, SEL1L		RETREG1, CALCOCO1	CCPG1, RTN3
GSE 45050	Cirrhotic vs. Healthy	MAN2B1, DERL3, RCN3	RNFT1, RNF139, RNF170, MAN1A2, UBXN8, EDEM1		—	ATL2, CCPG1, RETREG1, SEC62
GSE 11536	Advanced Fibrosis vs. Mild Fibrosis	INSIG1, MAN1A2, MAN2B1, UBE2G2	MAN1B1, BAG6, EDEM1, SEL1L, UBXN8, RNF5, XBP1, MAN2A2, UBE2K, RNF103, MAN1A1, STUB1, TRIM21, UBE2D1		—	CCPG1, SEC62, RTN3, CALCOCO2
**Analysis of primary or immortalized HSCs**						
**Dataset**	**Comparison**	**ERAD: Upregulated genes**		**ERAD: Downregulated genes**	**ER-phagy: Upregulated genes**	**ER-phagy: Downregulated genes**
GSE 68000	Primary hHSCs activated by stiffness	DNAJC10, RCN3, TRIM13, JKAMP, SEL1L, STT3B, ERLIN1, FAF2, EDEM1, ERLEC1, RHBDD1, HERPUD1, MAN1B1, UBE2K, NPLOC4, WFS1, RNF170, DNAJB14, DERL2, INSIG1, RNF185, UBE2N, UBE2D1		FBXO2, UBXN1, MAN2C1, RNF5, UBE2J1, MAN1C1, HSPA8	ATL3, RTN3	—
GSE 122710	LX-2 Cells: TGFβ vs Vehicle	DERL3, ERLEC1, DNAJC10, TRIM25, EDEM3, RNF103, RNF185, BAG6, TMEM129, SEL1L, DNAJC18, UBE2J1, JKAMP, UBXN1, AMFR		MAN1B1, VCP, UBE2N, HSPA8, WFS1, TRIM21, INSIG1, FBXO2	CCPG1, TEX264, RETREG1, ATL2, DDRGK1, CDK5RAP3/C53	—
GSE 151771	LX-2 Cells: TGFβ vs Vehicle	INSIG1, RNFT1, DNAJC18, UBE2K, RNF103, HERPUD1, UBE2D1, JKAMP, UBE2J1, DERL2, DNAJB11, UBE2G2, EDEM1, UBE2J2, STT3B, HSPA5, NPLOC4		FAF2, BAG6, UBXN1, TRIM25, UBXN6, SYVN1, TMEM129, MAN1A1, TMUB2, RNF139, RNF185, MAN1C1	CALCOCO2	ATL3, DDRGK1, TEX264, CDK5RAP3

In keeping with a protective role of ERAD in response to alcohol, *in vitro* studies have shown that ERAD inhibition sensitizes HepG2 cells to inflammation-induced cell death. In these studies, ERAD inhibition, or deletion of SEL1L increased ROS levels and disrupted mitochondrial morphology and function (Liu et al., 2020). Notably, HepG2 cells lack CYP2E1 expression, prompting the question of whether increased CYP2E1 expression in response to ERAD inhibition would protect these cells from the observed phenotypes. Indeed, E47 cells, HepG2 cells engineered to express CYP2E1, exhibited increased expression of nuclear factor-E2-related factor (Nrf2), a UPR-regulated transcription factor which increases expression of antioxidants, compared to parental HepG2 cells (Gong and Cederbaum, 2006). This may protect hepatocytes from oxidative stress caused by CYP2E1-mediated ethanol metabolism in combination with ERAD-mediated CYP2E1 degradation.

Finally, ERAD regulation of CYP2E1 may play a critical role in ALD progression outside of hepatocytes. The liver vasculature is essential for maintaining liver physiology, with changes in vascular tone or architecture leading to liver disease. Alcoholic liver disease is associated with dysregulation of liver sinusoidal endothelial cells (LSECs), and this may be related to ERAD dysfunction (Sarphie et al., 1997; Cohen and Nagy, 2011; Teschke, 2018). A recent study by Yang et al. showed that CYP2E1 expression increases in LSECs in response to ethanol, which in turn leads to acetylation of HSP90 (Yang et al., 2021b). Hsp90 is a cytoplasmic chaperone involved in protein folding but can also recruit proteins for ERAD (Giodini and Cresswell, 2008; Donnelly et al., 2013). This study focused on the role of HSP90 acetylation, which disrupts its interaction with eNOS. HSP90 protects eNOS from ERAD, thus promoting NO production. Upon pathogenic HSP90 acetylation, NO production decreases and liver injury worsens (Yang et al., 2021b). Deacetylation of HSP90 also protected mice from alcohol-induced injury in this study. Canonically, HSP90 acetylation disrupts its affinity for binding to client proteins, thus impairing effective folding, and potentially targeting them for ERAD. Based on the study in LSECs, CYP2E1 may directly dysregulate protein folding and targeting of substrates for ERAD by promoting HSP90 acetylation, in turn driving ALD pathogenesis.

In sum, these studies indicate that therapeutics aimed at promoting CYP2E1 turnover could limit ROS production and limit ethanol-induced damage.

### ERLAD in ALD

Autophagy and lysosomal degradation are tightly intertwined in ALD pathogenesis, but the contribution of ERLAD to ALD pathogenesis is unknown. Autophagy and lysosomal degradation protect hepatocytes from ethanol-mediated injury. Treatment of mice with rapamycin to promote autophagy limited injury in response to acute ethanol exposure, while overexpression of the transcription factor TFEB, which promotes lysosomal biogenesis, in mouse livers limited damage in a chronic ethanol feeding model (Ding et al., 2010; Chao et al., 2018). In turn, inhibition of autophagy or lysosomal degradation exacerbated ethanol-mediated liver injury. While little is known regarding the contribution of ERLAD to ALD progression, there is evidence that these processes are involved.

One potential role for ERLAD is through degradation of CYP2E1. Alcohol consumption, and subsequent ROS production by CYP2E1, increases autophagic flux (Kim and Kim, 2020; Chen et al., 2021). This increase is associated with removal of organelles such as mitochondria damaged by ROS, as well as in response to ER stress caused by ROS and hepatocyte dysfunction. Studies have also shown that CYP2E1 can undergo autophagic degradation in hepatocytes. This could have a critical impact on ethanol-mediated liver injury, as CYP2E1 and ethanol exposure is linked to reduced autophagy, potentially stabilizing CYP2E1 and further propagating liver damage. Our analysis of RNAseq databases revealed upregulation of ATL3 and DDRGK1 in patients with alcoholic hepatitis, as well as CCPG1 in patients with alcoholic steatosis compared to mild hepatitis (Table 2) (Caillot et al., 2009a; Bourd-Boittin et al., 2011; Affo et al., 2013; Trepo et al., 2018; Hyun et al., 2020). Modification of damaged CYP proteins by ubiquitination or UFMylation could target them for ERLAD by p62 or DDRGK1 respectively. p62, known for its role in autophagy, was recently reported to target ER membrane proteins for ERLAD through ubiquitination and may serve such a role in hepatocytes when ERAD is overwhelmed or inefficient at degrading CYP2E1, but this hypothesis requires further testing.

Based on the data presented above, enhanced protein degradation through ERAD or ERLAD could limit ethanol-induced hepatocyte damage and progression of ALD.

## ER Quality Control Pathways in Fibrogenesis

A hallmark of the progression of chronic liver disease is fibrogenesis. Sustained injury to the liver promotes inflammation and activation of hepatic stellate cells (HSCs), the primary fibrogenic cell in the liver. Upon activation, HSCs produce fibrogenic proteins including procollagen isoforms I, III, VI, fibronectin, and numerous other proteins destined for secretion. Increased fibrogenic secretion requires ER expansion, increased expression of chaperone proteins, and increased ER quality control to facilitate degradation of misfolded proteins. Indeed, activated HSCs exhibit increased UPR signaling. Both IRE1α and ATF6α, upstream regulators of ERAD and autophagy, are crucial for hepatic fibrogenesis *in vivo*, though these studies have focused on transcriptional regulation of HSC activation and fibrogenesis downstream of the UPR, and not on ER quality control pathways (Xue et al., 2021; (Hernandez-Gea et al., 2013; Heindryckx et al., 2016; Liu et al., 2019). PERK, also known for a role in autophagy, promotes fibrogenesis (Koo et al., 2016; Zheng et al., 2019; B’Chir et al., 2013; Kang et al., 2017). With both IRE1α and ATF6α pathways elevated in fibrogenic HSCs, it is likely that ERAD and ERLAD also contribute to HSC activation and fibrogenesis.

The connection between ER quality control pathways and fibrogenesis are only beginning to be understood. Analysis of whole liver RNAseq from patients with advanced fibrosis or cirrhosis revealed a general downregulation of ERAD-associated genes and ERLAD-associated genes (Table 3) (Caillot et al., 2009b; Mas et al., 2009; Darpolor et al., 2014). As HSCs only make up a small fraction of the liver, we also analyzed RNAseq performed on primary human or immortalized human HSCs (LX-2 cells). These analyses overwhelmingly indicated upregulation of ERAD- and ERLAD-associated genes during HSC activation (Caillot et al., 2009b; Mas et al., 2009; Darpolor et al., 2014). The upregulation of ER-phagy receptors fits with recent studies in osteoblasts from zebrafish where FAM134B facilitates lysosomal degradation of misfolded or overexpressed procollagen Ia1 and 1a2 (Forrester et al., 2019). This role for FAM134B was later confirmed in mouse embryonic fibroblasts (MEFs) for a misfolded procollagen II mutant. Targeting misfolded procollagen I for degradation involved both calnexin and UGGT1 ((Fregno et al., 2021). Increased ERLAD-mediated degradation in activated HSCs could promote fibrogenesis through relieving the ER burden of misfolded procollagen I, thus limiting ER stress and promoting HSC survival. Whether this role is conserved in HSCs, and its potential for targeting *in vivo,* are unknown. Preliminary data from the Maiers lab indicates that ER-phagic flux increased in activated HSCs, and this increase is dependent on ATF6α signaling (unpublished observation). We are currently studying the roles of specific ER-phagy receptors in HSC activation and fibrogenesis, and potential targetability of these proteins to limit liver fibrosis.

## Therapeutic Targeting of Protein Degradation Pathways

Dysregulated protein degradation is a hallmark of several diseases and has been a focus of therapeutic strategies for decades. Inhibition of proteasomal degradation successfully limits multiple myeloma progression through increasing cellular stress and driving apoptosis of cancer cells. The approach of proteasomal inhibition may be attractive if cell death is the optimal endpoint such as with removal of fibrogenic HSCs. Unfortunately, hepatocyte death is a critical driver of chronic liver disease which limits the use of a general proteasomal inhibitor. The potential for activating or inhibiting a subset of proteasomal or lysosomal degradation, such as ERAD or ERLAD could provide nuanced targeting that allows to reduced stress and injury without activating cell death pathways.

### Targeting Protein Degradation in AATD

Alpha-1 antitrypsin deficiency (AATD) induces liver disease by the increased accumulation of misfolded AAT soluble and insoluble aggregates in the ER of hepatocytes. Given this, targeting ER-dependent protein degradation in AATD to alleviate the liver disease component of this illness specifically, seems a good therapeutic strategy.

The role of autophagy and macro-ER-phagy in PI-Z degradation led to numerous groups testing autophagic drugs in order to reduce hepatocyte aggregates and reduce the liver burden of AATD. Drugs that positively regulate autophagy, such as carbamazepine (CBZ), rapamycin, ezetimibe, nor-ursodeoxycholic acid (norUDCA), or glibenclamide, have been tested in AATD models and have demonstrated promising and significant results in decreasing the accumulation of PI-Z (Hidvegi et al., 2010; Kaushal et al., 2010; Yamamura et al., 2014; Tang et al., 2016; Tang et al., 2018; Wang et al., 2019). Hidvegi and colleagues demonstrated that CZB could directly increase autophagy of both insoluble and soluble PI-Z aggregates (Hidvegi et al., 2010). Furthermore mice treated with CZB for 2 weeks at a daily dose of 250 mg/kg had significantly increased autophagy of PI-Z, which directly decreased liver disease and fibrosis. Another group, giving weekly rapamycin for 12 weeks at a dose of 10 mg/kg observed a significant increase in autophagic activity as evident by decreased PI-Z aggregates within the liver (Kaushal et al., 2010). They also found reduced liver fibrosis with rapamycin treatment compared to non-treated mice. A third group tried short-term ezetimibe treatment in human primary hepatocytes, which induced significant autophagy as evidenced by increased LC3-II and decreased p62 and p-S6K (Yamamura et al., 2014). They further demonstrated that PI-Z diminished in an ezetimibe dose-dependent fashion. In recent years bile acids have gained traction as great therapeutics with little side effects, one group tried treating both AATD mice and PI-Z transfected HTOZ cells with norUDCA (Tang et al., 2016; Tang et al., 2018). The *in vitro* treatment resulted in increased autophagy of PI-Z protein aggregates in a dose-dependent manner. PI-Z mice treated with norUDCA at a daily dose of 425 mg/kg for 6 weeks developed significantly less liver disease as evidenced by diminished ALT levels, steatosis, and inflammatory foci. Finally Wang et al. screened PI-Z mutant *C. elegans* for potential therapeutics, discovering that glibenclamide increased autophagy of PI-Z aggregates (Wang et al., 2019). They initially tested the finding in a PI-Z expressing HTOZ cell line. Cells demonstrated an increase in autophagy of both soluble and insoluble PI-Z in a dose-dependent manner (1–100 μM). The group then tested analogs of glibenclamide and discovered they too had significant autophagic potential in a PI-Z mouse model and culminated in decreased fibrosis.

Excitingly, the aforementioned work informed the development of a clinical trial. David Perlmutter’s group has undertaken a clinical trial with carbamazepine (CZB) in AATD patients. The results remain unknown as the study just completed, one could easily imagine the positive impact increasing autophagy in AATD could have to reduce liver injury and fibrosis in this patient population. While this is the only clinical trial currently investigating autophagy in AATD, of the 124 registered clinical trials for patients with AATD at the time this review was written, it seems further investigating ERLAD and even ERAD during AATD would be an ideal strategy to combat at least the liver disease portion of the disease, even though it would not help alleviate the lung portion.

### Targeting Protein Degradation in NAFLD

There are over a thousand registered clinical trials studying NAFLD. This is unsurprising given the front stage in the therapeutic world this disease has taken in the past decade; however, it is surprising that not one trial appears to directly investigate ER-dependent protein or lipid degradation. Even autophagy, which plays a critical pathogenic role in NAFLD/NASH is not being investigated in patients. When reviewing results in mouse studies using autophagy inducing drugs (Liu et al., 2009; Lin et al., 2013), the studies were extremely promising. In a mouse model of HFD feeding for 12 weeks mice were given CBZ at 25 mg/kg dose or rapamycin at 2 mg/kg dose every other day for the last week of feeding. The treatment significantly reduced lipid droplet accumulation, hepatic and serum triglycerides, and plasma insulin. Though no difference was seen between treated and untreated controls in ALT levels (Lin et al., 2013). However, given the safety profile of CZB in the numerous neurological diseases in which it has been studied one must wonder why this drug has not been tested in the NAFLD patient population, making this truly an unmet need. NAFLD is a complex metabolic disease with multiple facets that can be targeted and given the role ER stress plays in this disease ER-dependent degradation pathways, which would alleviate ER stress, are ideal targets.

### Targeting Protein Degradation in ALD

Increasing lysosomal or proteasomal degradation in ALD is a promising strategy that is understudied. Over a decade past, murine studies showed that increased autophagy, achieved through rapamycin treatment, limited the toxicity of acute ethanol exposure (Ding et al., 2010). This was attributed to enhanced degradation of lipid droplets, serving to restore lipid homeostasis in hepatocytes and limit steatosis. Subsequent studies have also suggested that autophagy protects the liver from chronic ethanol exposure (Lin et al., 2013; Lu and Cederbaum, 2015). Currently, there are no clinical trials targeting protein degradation in patients with ALD, due in part to the unclear regulation of autophagy in response to alcohol. Recent studies aimed to understand whether activation of TFEB could limit alcohol-induced liver injury. While activation of TFEB through administration of Trehalose increased autophagic flux *in vitro*, it failed to limit alcohol-induced liver injury *in vivo* (Chao et al., 2021). Targeting mTOR to increase autophagy has also been proposed as a potential mechanism to activate autophagy in patients ALD (extensively reviewed elsewhere), but its broad impact on autophagy and lysosomal degradation could impact a wide breadth of processes (Kim and Kim, 2020; Flessa et al., 2021; Williams and Ding, 2020; Zhou et al., 2021). Specific activation of protein degradation pathways, such as ERLAD, could provide a more targeted approach for patients with ALD.

### Targeting Protein Degradation in Liver Fibrosis

The secretion of procollagen I and other fibrogenic proteins is a widely pursued studied antifibrotic strategy. Several groups have sought to target degradative mechanisms in hepatic and extrahepatic disease through inhibiting the proteosome or autophagy. Bortezomib, a proteosome inhibitor used to treat Multiple Myeloma, reduces liver fibrosis in cholestatic mouse models (MDR2^−/−^ Bile duct ligation), as well as renal and lung fibrosis in other mouse models (Anan et al., 2006a; Jalan-Sakrikar et al., 2019; Zhou et al., 2019; Penke et al., 2021). The mechanisms associated with the fibrosis reduction differ, with focus on EZH2, TGFβ signaling, or limiting fibroblast activation, but the anti-fibrotic results were similar. *In vitro* studies further found that proteosome inhibition leads to HSC apoptosis (Anan et al., 2006b)). These findings highlight the importance of proteasomal degradation in HSCs and fibrogenesis, but no studies have directly studied the impact of ERAD on fibrogenesis. ER stress and IRE1α signaling are elevated in activated HSCs, while disruption of IRE1α or ATF6α signaling in HSCs limits their activation and fibrosis in mice (Xue et al., 2021; Hernandez-Gea et al., 2013; Heindryckx et al., 2016; Liu et al., 2019). As IRE1α is a critical regulator of ERAD, and either proteasome inhibition or IRE1α inhibition limits HSC activation and fibrogenesis, the potential contributions of ERAD to these processes merit further study.

Regulation of autophagy has also been studied to target fibrogenesis. The autophagy inhibitor 3-MA reduced CCl_4_-mediated fibrosis through promoting HSC apoptosis through the NF-kB pathway, while inhibition of autophagy using different autophagic inhibitors similarly limited HSC activation and fibrosis ((Wang et al., 2017). Other studies indicate that autophagy is protective in HSCs, with autophagy activation limiting HSC activation by TGFβ, and reducing fibrosis (Hidvegi et al., 2010; Zhu et al., 1999; Bridle et al., 2009; Xie et al., 2018). We will not expand on the dichotomous role of autophagy in HSC activation and fibrogenesis, as it is well discussed in recent reviews (Lucantoni et al., 2021; Sun et al., 2021). The major point that we hope to make is that ERLAD pathways could serve as a unique, targetable form of autophagy that directly impacts procollagen I by tipping the balance between procollagen I degradation and secretion in HSCs. Investigating the role of ERLAD in HSC activation is crucial for understanding 1) the nuances of autophagic regulation in fibrogenesis, 2) how HSCs accommodate the burden of increased procollagen I degradation, and 3) potential strategies for targeting this process.

## Conclusion

ER Quality Control pathways are important, targetable processes which are understudied in the liver. Review of the literature and analysis of publicly available datasets clearly show that these processes are dysregulated in patients with chronic liver disease; however, their contribution to pathogenesis remains unclear. Advancements in technology such as mass spectrometry (e.g., thermal proteome profiling), drug design, high resolution microscopy, and others will allow for careful and systematic investigation of these pathways in liver physiology, different hepatic cell lineages, and under pathological conditions. These studies should provide crucial insight into an understudied area of liver physiology, and identify targetable mechanisms for limiting liver injury and disease progression.

## References

[B1] Affo S. Dominguez M. Lozano J. J. Sancho-Bru P. Rodrigo-Torres D. Morales-Ibanez O. (2013). Transcriptome Analysis Identifies TNF Superfamily Receptors as Potential Therapeutic Targets in Alcoholic Hepatitis. Gut 62 (3), 452–460. 10.1136/gutjnl-2011-301146 22637703 PMC4064940

[B2] Ahrens M. Ammerpohl O. von Schonfels W. Kolarova J. Bens S. Itzel T. (2013). DNA Methylation Analysis in Nonalcoholic Fatty Liver Disease Suggests Distinct Disease-specific and Remodeling Signatures after Bariatric Surgery. Cell Metab 18 (2), 296–302. 10.1016/j.cmet.2013.07.004 23931760

[B3] An H. Ordureau A. Paulo J. A. Shoemaker C. J. Denic V. Harper J. W. (2019). TEX264 Is an Endoplasmic Reticulum-Resident ATG8-Interacting Protein Critical for ER Remodeling during Nutrient Stress. Mol. Cell 74 (5), 891–908. 10.1016/j.molcel.2019.03.034 31006537 PMC6747008

[B4] Anan A. Baskin-Bey E. S. Bronk S. F. Werneburg N. W. Shah V. H. Gores G. J. (2006). Proteasome Inhibition Induces Hepatic Stellate Cell Apoptosis. Hepatology 43 (2), 335–344. 10.1002/hep.21036 16440346

[B5] Anan A. Baskin-Bey E. S. Isomoto H. Mott J. L. Bronk S. F. Albrecht J. H. (2006). Proteasome Inhibition Attenuates Hepatic Injury in the Bile Duct-Ligated Mouse. Am. J. Physiol. Gastrointest. Liver Physiol. 291 (4), G709–G716. 10.1152/ajpgi.00126.2006 16798723

[B6] Arendt B. M. Comelli E. M. Ma D. W. Lou W. Teterina A. Kim T. (2015). Altered Hepatic Gene Expression in Nonalcoholic Fatty Liver Disease Is Associated with Lower Hepatic N-3 and N-6 Polyunsaturated Fatty Acids. Hepatology 61 (5), 1565–1578. 10.1002/hep.27695 25581263

[B7] Asrani S. K. Devarbhavi H. Eaton J. Kamath P. S. (2019). Burden of Liver Diseases in the World. J. Hepatol. 70 (1), 151–171. 10.1016/j.jhep.2018.09.014 30266282

[B8] B'Chir W. Maurin A. C. Carraro V. Averous J. Jousse C. Muranishi Y. (2013). The eIF2alpha/ATF4 Pathway Is Essential for Stress-Induced Autophagy Gene Expression. Nucleic Acids Res. 41 (16), 7683–7699. 10.1093/nar/gkt563 23804767 PMC3763548

[B9] Baiceanu A. Mesdom P. Lagouge M. Foufelle F. (2016). Endoplasmic Reticulum Proteostasis in Hepatic Steatosis. Nat. Rev. Endocrinol. 12 (12), 710–722. 10.1038/nrendo.2016.124 27516341

[B10] Baker S. S. Baker R. D. Liu W. Nowak N. J. Zhu L. (2010). Role of Alcohol Metabolism in Non-alcoholic Steatohepatitis. PLoS One 5 (3), e9570. 10.1371/journal.pone.0009570 20221393 PMC2833196

[B11] Ballinger C. A. Connell P. Wu Y. Hu Z. Thompson L. J. Yin L. Y. (1999). Identification of CHIP, a Novel Tetratricopeptide Repeat-Containing Protein that Interacts with Heat Shock Proteins and Negatively Regulates Chaperone Functions. Mol. Cell Biol 19 (6), 4535–4545. 10.1128/mcb.19.6.4535 10330192 PMC104411

[B12] Barak A. J. Beckenhauer H. C. Tuma D. J. (1996). Betaine Effects on Hepatic Methionine Metabolism Elicited by Short-Term Ethanol Feeding. Alcohol 13 (5), 483–486. 10.1016/0741-8329(96)00040-7 8888945

[B13] Bhattacharya A. Sun S. Wang H. Liu M. Long Q. Yin L. (2018). Hepatic Sel1L-Hrd1 ER-Associated Degradation (ERAD) Manages FGF21 Levels and Systemic Metabolism via CREBH. EMBO J. 37 (22). 10.15252/embj.201899277 PMC6236331 30389665

[B14] Boren J. Packard C. J. Taskinen M. R. (2020). The Roles of ApoC-III on the Metabolism of Triglyceride-Rich Lipoproteins in Humans. Front. Endocrinol. (Lausanne) 11, 474. 10.3389/fendo.2020.00474 32849270 PMC7399058

[B15] Bourd-Boittin K. Bonnier D. Leyme A. Mari B. Tuffery P. Samson M. (2011). Protease Profiling of Liver Fibrosis Reveals the ADAM Metallopeptidase with Thrombospondin Type 1 Motif, 1 as a central Activator of Transforming Growth Factor Beta. Hepatology 54 (6), 2173–2184. 10.1002/hep.24598 21826695

[B16] Bridle K. R. Popa C. Morgan M. L. Sobbe A. L. Clouston A. D. Fletcher L. M. (2009). Rapamycin Inhibits Hepatic Fibrosis in Rats by Attenuating Multiple Profibrogenic Pathways. Liver Transpl. 15 (10), 1315–1324. 10.1002/lt.21804 19790156

[B17] Brodsky J. L. Fisher E. A. (2008). The many Intersecting Pathways Underlying Apolipoprotein B Secretion and Degradation. Trends Endocrinol. Metab. 19 (7), 254–259. 10.1016/j.tem.2008.07.002 18691900 PMC3216472

[B18] Caillot F. Derambure C. Bioulac-Sage P. Francois A. Scotte M. Goria O. (2009). Transient and Etiology-Related Transcription Regulation in Cirrhosis Prior to Hepatocellular Carcinoma Occurrence. World J. Gastroenterol. 15 (3), 300–309. 10.3748/wjg.15.300 19140229 PMC2653326

[B19] Caillot F. Hiron M. Goria O. Gueudin M. Francois A. Scotte M. (2009). Novel Serum Markers of Fibrosis Progression for the Follow-Up of Hepatitis C Virus-Infected Patients. Am. J. Pathol. 175 (1), 46–53. 10.2353/ajpath.2009.080850 19477948 PMC2708793

[B20] Cazanave S. C. Elmi N. A. Akazawa Y. Bronk S. F. Mott J. L. Gores G. J. (2010). CHOP and AP-1 Cooperatively Mediate PUMA Expression during Lipoapoptosis. Am. J. Physiol. Gastrointest. Liver Physiol. 299 (1), G236–G243. 10.1152/ajpgi.00091.2010 20430872 PMC2904106

[B21] Cazanave S. C. Mott J. L. Bronk S. F. Werneburg N. W. Fingas C. D. Meng X. W. (2011). Death Receptor 5 Signaling Promotes Hepatocyte Lipoapoptosis. J. Biol. Chem. 286 (45), 39336–39348. 10.1074/jbc.m111.280420 21941003 PMC3234758

[B22] Chao X. Wang S. Yang L. Ni H. M. Ding W. X. (2021). Trehalose Activates Hepatic Transcription Factor EB (TFEB) but Fails to Ameliorate Alcohol-Impaired TFEB and Liver Injury in Mice. Alcohol. Clin. Exp. Res. 45 (10), 1950–1964. 10.1111/acer.14695 34486131 PMC8602756

[B23] Chao X. Wang S. Zhao K. Li Y. Williams J. A. Li T. (2018). Impaired TFEB-Mediated Lysosome Biogenesis and Autophagy Promote Chronic Ethanol-Induced Liver Injury and Steatosis in Mice. Gastroenterology 155 (3), 865–879 e12. 10.1053/j.gastro.2018.05.027 29782848 PMC6120772

[B24] Chen C. Wang S. Yu L. Mueller J. Fortunato F. Rausch V. (2021). H2O2-mediated Autophagy during Ethanol Metabolism. Redox Biol. 46, 102081. 10.1016/j.redox.2021.102081 34343907 PMC8350071

[B25] Chen Q. Xiao Y. Chai P. Zheng P. Teng J. Chen J. (2019). ATL3 Is a Tubular ER-Phagy Receptor for GABARAP-Mediated Selective Autophagy. Curr. Biol. 29 (5), 846–855. 10.1016/j.cub.2019.01.041 30773365

[B26] Chen W. Y. Zhang J. Ghare S. Barve S. McClain C. Joshi-Barve S. (2016). Acrolein Is a Pathogenic Mediator of Alcoholic Liver Disease and the Scavenger Hydralazine Is Protective in Mice. Cell Mol Gastroenterol Hepatol 2 (5), 685–700. 10.1016/j.jcmgh.2016.05.010 28119953 PMC5042858

[B27] Chen X. Zhang F. Gong Q. Cui A. Zhuo S. Hu Z. (2016). Hepatic ATF6 Increases Fatty Acid Oxidation to Attenuate Hepatic Steatosis in Mice through Peroxisome Proliferator-Activated Receptor Alpha. Diabetes 65 (7), 1904–1915. 10.2337/db15-1637 27207533

[B28] Chino H. Hatta T. Natsume T. Mizushima N. (2019). Intrinsically Disordered Protein TEX264 Mediates ER-Phagy. Mol. Cell 74 (5), 909–921. 10.1016/j.molcel.2019.03.033 31006538

[B29] Choi K. Kim H. Kang H. Lee S. Y. Lee S. J. Back S. H. (2014). Regulation of Diacylglycerol Acyltransferase 2 Protein Stability by Gp78-Associated Endoplasmic-Reticulum-Associated Degradation. FEBS J. 281 (13), 3048–3060. 10.1111/febs.12841 24820123

[B30] Christianson J. C. Shaler T. A. Tyler R. E. Kopito R. R. (2008). OS-9 and GRP94 Deliver Mutant Alpha1-Antitrypsin to the Hrd1-Sel1l Ubiquitin Ligase Complex for ERAD. Nat. Cell Biol 10 (3), 272–282. 10.1038/ncb1689 18264092 PMC2757077

[B31] Cinque L. De Leonibus C. Iavazzo M. Krahmer N. Intartaglia D. Salierno F. G. (2020). MiT/TFE Factors Control ER-Phagy via Transcriptional Regulation of FAM134B. EMBO J. 39 (17), e105696. 10.15252/embj.2020105696 32716134 PMC7459426

[B32] Cohen J. I. Nagy L. E. (2011). Pathogenesis of Alcoholic Liver Disease: Interactions between Parenchymal and Non-parenchymal Cells. J. Dig. Dis. 12 (1), 3–9. 10.1111/j.1751-2980.2010.00468.x 21091930 PMC5061145

[B33] Connell P. Ballinger C. A. Jiang J. Wu Y. Thompson L. J. Hohfeld J. (2001). The Co-chaperone CHIP Regulates Protein Triage Decisions Mediated by Heat-Shock Proteins. Nat. Cell Biol 3 (1), 93–96. 10.1038/35050618 11146632

[B34] Correia M. A. Wang Y. Kim S. M. Guan S. (2014). Hepatic Cytochrome P450 Ubiquitination: Conformational Phosphodegrons for E2/E3 Recognition? IUBMB Life 66 (2), 78–88. 10.1002/iub.1247 24488826 PMC3959620

[B35] Darpolor M. M. Basu S. S. Worth A. Nelson D. S. Clarke-Katzenberg R. H. Glickson J. D. (2014). The Aspartate Metabolism Pathway Is Differentiable in Human Hepatocellular Carcinoma: Transcriptomics and (13) C-Isotope Based Metabolomics. NMR Biomed. 27 (4), 381–389. 10.1002/nbm.3072 24497316 PMC3962779

[B36] Dasgupta D. Nakao Y. Mauer A. S. Thompson J. M. Sehrawat T. S. Liao C. Y. (2020). IRE1A Stimulates Hepatocyte-Derived Extracellular Vesicles that Promote Inflammation in Mice with Steatohepatitis. Gastroenterology 159 (4), 1487–1503. 10.1053/j.gastro.2020.06.031 32574624 PMC7666601

[B37] Deng Y. Wang Z. V. Tao C. Gao N. Holland W. L. Ferdous A. (2013). The Xbp1s/GalE axis Links ER Stress to Postprandial Hepatic Metabolism. J. Clin. Invest. 123 (1), 455–468. 10.1172/jci62819 23257357 PMC3533268

[B38] Ding W. X. Li M. Chen X. Ni H. M. Lin C. W. Gao W. (2010). Autophagy Reduces Acute Ethanol-Induced Hepatotoxicity and Steatosis in Mice. Gastroenterology 139 (5), 1740–1752. 10.1053/j.gastro.2010.07.041 20659474 PMC4129642

[B39] Donnelly B. F. Needham P. G. Snyder A. C. Roy A. Khadem S. Brodsky J. L. (2013). Hsp70 and Hsp90 Multichaperone Complexes Sequentially Regulate Thiazide-Sensitive Cotransporter Endoplasmic Reticulum-Associated Degradation and Biogenesis. J. Biol. Chem. 288 (18), 13124–13135. 10.1074/jbc.m113.455394 23482560 PMC3642353

[B40] Doody E. E. Groebner J. L. Walker J. R. Frizol B. M. Tuma D. J. Fernandez D. J. (2017). Ethanol Metabolism by Alcohol Dehydrogenase or Cytochrome P450 2E1 Differentially Impairs Hepatic Protein Trafficking and Growth Hormone Signaling. Am. J. Physiol. Gastrointest. Liver Physiol. 313 (6), G558–G69. 10.1152/ajpgi.00027.2017 28864499 PMC5814672

[B41] Du J. Ji Y. Qiao L. Liu Y. Lin J. (2020). Cellular Endo-Lysosomal Dysfunction in the Pathogenesis of Non-alcoholic Fatty Liver Disease. Liver Int. 40 (2), 271–280. 10.1111/liv.14311 31765080

[B42] Duwaerts C. C. Siao K. Soon R. K. Jr. Her C. Iwawaki T. Kohno K. (2021). Hepatocyte-specific Deletion of XBP1 Sensitizes Mice to Liver Injury through Hyperactivation of IRE1alpha. Cell Death Differ 28 (5), 1455–1465. 10.1038/s41418-020-00671-1 33219328 PMC8166833

[B43] Feng L. Zhang J. Zhu N. Ding Q. Zhang X. Yu J. (2017). Ubiquitin Ligase SYVN1/HRD1 Facilitates Degradation of the SERPINA1 Z Variant/alpha-1-Antitrypsin Z Variant via SQSTM1/p62-dependent Selective Autophagy. Autophagy 13 (4), 686–702. 10.1080/15548627.2017.1280207 28121484 PMC5388218

[B44] Fernandez A. Matias N. Fucho R. Ribas V. Von Montfort C. Nuno N. (2013). ASMase Is Required for Chronic Alcohol Induced Hepatic Endoplasmic Reticulum Stress and Mitochondrial Cholesterol Loading. J. Hepatol. 59 (4), 805–813. 10.1016/j.jhep.2013.05.023 23707365 PMC3779525

[B45] Fisher E. A. Zhou M. Mitchell D. M. Wu X. Omura S. Wang H. (1997). The Degradation of Apolipoprotein B100 Is Mediated by the Ubiquitin-Proteasome Pathway and Involves Heat Shock Protein 70. J. Biol. Chem. 272 (33), 20427–20434. 10.1074/jbc.272.33.20427 9252351

[B46] Flessa C.-M. Kyrou I. Nasiri-Ansari N. Kaltsas G. Papavassiliou A. G. Kassi E. (2021). Endoplasmic Reticulum Stress and Autophagy in the Pathogenesis of Non-alcoholic Fatty Liver Disease (NAFLD): Current Evidence and Perspectives. Curr. Obes. Rep. 10 (2), 134–161. 10.1007/s13679-021-00431-3 33751456

[B47] Forrester A. De Leonibus C. Grumati P. Fasana E. Piemontese M. Staiano L. (2019). A Selective ER-Phagy Exerts Procollagen Quality Control via a Calnexin-Fam134b Complex. EMBO J. 38 (2). 10.15252/embj.201899847 PMC6331724 30559329

[B48] Fregno I. Fasana E. Bergmann T. J. Raimondi A. Loi M. Solda T. (2018). ER-to-lysosome-associated Degradation of Proteasome-Resistant ATZ Polymers Occurs via Receptor-Mediated Vesicular Transport. EMBO J. 37 (17). 10.15252/embj.201899259 PMC6120659 30076131

[B49] Fregno I. Fasana E. Solda T. Galli C. Molinari M. (2021). N-glycan Processing Selects ERAD-Resistant Misfolded Proteins for ER-To-Lysosome-Associated Degradation. EMBO J. 40 (15), e107240. 10.15252/embj.2020107240 34152647 PMC8327951

[B50] Fregno I. Molinari M. (2018). Endoplasmic Reticulum Turnover: ER-Phagy and Other Flavors in Selective and Non-selective ER Clearance. F1000Res 7, 454. 10.12688/f1000research.13968.1 29744037 PMC5904726

[B51] Fregno I. Molinari M. (2019). Proteasomal and Lysosomal Clearance of Faulty Secretory Proteins: ER-Associated Degradation (ERAD) and ER-To-Lysosome-Associated Degradation (ERLAD) Pathways. Crit. Rev. Biochem. Mol. Biol. 54 (2), 153–163. 10.1080/10409238.2019.1610351 31084437

[B52] French S. W. Masouminia M. Samadzadeh S. Tillman B. C. Mendoza A. French B. A. (2017). Role of Protein Quality Control Failure in Alcoholic Hepatitis Pathogenesis. Biomolecules 7 (1). 10.3390/biom7010011 PMC5372723 28208700

[B53] Fujii J. Homma T. Kobayashi S. Seo H. G. (2018). Mutual Interaction between Oxidative Stress and Endoplasmic Reticulum Stress in the Pathogenesis of Diseases Specifically Focusing on Non-alcoholic Fatty Liver Disease. World J. Biol. Chem. 9 (1), 1–15. 10.4331/wjbc.v9.i1.1 30364769 PMC6198288

[B54] Fumagalli F. Noack J. Bergmann T. J. Cebollero E. Pisoni G. B. Fasana E. (2016). Translocon Component Sec62 Acts in Endoplasmic Reticulum Turnover during Stress Recovery. Nat. Cell Biol 18 (11), 1173–1184. 10.1038/ncb3423 27749824

[B55] Gelling C. L. Dawes I. W. Perlmutter D. H. Fisher E. A. Brodsky J. L. (2012). The Endosomal Protein-Sorting Receptor Sortilin Has a Role in Trafficking Alpha-1 Antitrypsin. Genetics 192 (3), 889–903. 10.1534/genetics.112.143487 22923381 PMC3522165

[B56] Giodini A. Cresswell P. (2008). Hsp90-mediated Cytosolic Refolding of Exogenous Proteins Internalized by Dendritic Cells. EMBO J. 27 (1), 201–211. 10.1038/sj.emboj.7601941 18046456 PMC2104710

[B57] Gong P. Cederbaum A. I. (2006). Nrf2 Is Increased by CYP2E1 in Rodent Liver and HepG2 Cells and Protects against Oxidative Stress Caused by CYP2E1. Hepatology 43 (1), 144–153. 10.1002/hep.21004 16374848

[B58] Gonzalez-Rodriguez A. Mayoral R. Agra N. Valdecantos M. P. Pardo V. Miquilena-Colina M. E. (2014). Impaired Autophagic Flux Is Associated with Increased Endoplasmic Reticulum Stress during the Development of NAFLD. Cell Death Dis 5, e1179. 10.1038/cddis.2014.162 24743734 PMC4001315

[B59] Greenblatt E. J. Olzmann J. A. Kopito R. R. (2011). Derlin-1 Is a Rhomboid Pseudoprotease Required for the Dislocation of Mutant Alpha-1 Antitrypsin from the Endoplasmic Reticulum. Nat. Struct. Mol. Biol. 18 (10), 1147–1152. 10.1038/nsmb.2111 21909096 PMC3196324

[B60] Grumati P. Dikic I. Stolz A. (2018). ER-phagy at a Glance. J. Cell Sci 131 (17). 10.1242/jcs.217364 30177506

[B61] Grumati P. Morozzi G. Holper S. Mari M. Harwardt M. I. Yan R. (2017). Full Length RTN3 Regulates Turnover of Tubular Endoplasmic Reticulum via Selective Autophagy. Elife 6. 10.7554/eLife.25555 PMC5517149 28617241

[B62] Gusarova V. Caplan A. J. Brodsky J. L. Fisher E. A. (2001). Apoprotein B Degradation Is Promoted by the Molecular Chaperones Hsp90 and Hsp70. J. Biol. Chem. 276 (27), 24891–24900. 10.1074/jbc.m100633200 11333259

[B63] Haze K. Yoshida H. Yanagi H. Yura T. Mori K. (1999). Mammalian Transcription Factor ATF6 Is Synthesized as a Transmembrane Protein and Activated by Proteolysis in Response to Endoplasmic Reticulum Stress. Mol. Biol. Cell 10 (11), 3787–3799. 10.1091/mbc.10.11.3787 10564271 PMC25679

[B64] Heindryckx F. Binet F. Ponticos M. Rombouts K. Lau J. Kreuger J. (2016). Endoplasmic Reticulum Stress Enhances Fibrosis through IRE1alpha-Mediated Degradation of miR-150 and XBP-1 Splicing. EMBO Mol. Med. 8 (7), 729–744. 10.15252/emmm.201505925 27226027 PMC4931288

[B65] Hernandez-Gea V. Hilscher M. Rozenfeld R. Lim M. P. Nieto N. Werner S. (2013). Endoplasmic Reticulum Stress Induces Fibrogenic Activity in Hepatic Stellate Cells through Autophagy. J. Hepatol. 59 (1), 98–104. 10.1016/j.jhep.2013.02.016 23485523 PMC3686909

[B66] Hetz C. Zhang K. Kaufman R. J. (2020). Mechanisms, Regulation and Functions of the Unfolded Protein Response. Nat. Rev. Mol. Cell Biol 21 (8), 421–438. 10.1038/s41580-020-0250-z 32457508 PMC8867924

[B67] Hidvegi T. Ewing M. Hale P. Dippold C. Beckett C. Kemp C. (2010). An Autophagy-Enhancing Drug Promotes Degradation of Mutant Alpha1-Antitrypsin Z and Reduces Hepatic Fibrosis. Science 329 (5988), 229–232. 10.1126/science.1190354 20522742

[B68] Howarth D. L. Lindtner C. Vacaru A. M. Sachidanandam R. Tsedensodnom O. Vasilkova T. (2014). Activating Transcription Factor 6 Is Necessary and Sufficient for Alcoholic Fatty Liver Disease in Zebrafish. Plos Genet. 10 (5), e1004335. 10.1371/journal.pgen.1004335 24874946 PMC4038464

[B69] Hrizo S. L. Gusarova V. Habiel D. M. Goeckeler J. L. Fisher E. A. Brodsky J. L. (2007). The Hsp110 Molecular Chaperone Stabilizes Apolipoprotein B from Endoplasmic Reticulum-Associated Degradation (ERAD). J. Biol. Chem. 282 (45), 32665–32675. 10.1074/jbc.m705216200 17823116 PMC2666968

[B70] Hwang J. Qi L. (2018). Quality Control in the Endoplasmic Reticulum: Crosstalk between ERAD and UPR Pathways. Trends Biochem. Sci. 43 (8), 593–605. 10.1016/j.tibs.2018.06.005 30056836 PMC6327314

[B71] Hyun J. Sun Z. Ahmadi A. R. Bangru S. Chembazhi U. V. Du K. (2020). Epithelial Splicing Regulatory Protein 2-mediated Alternative Splicing Reprograms Hepatocytes in Severe Alcoholic Hepatitis. J. Clin. Invest. 130 (4), 2129–2145. 10.1172/jci132691 31945016 PMC7108908

[B72] Iida Y. Fujimori T. Okawa K. Nagata K. Wada I. Hosokawa N. (2011). SEL1L Protein Critically Determines the Stability of the HRD1-Sel1l Endoplasmic Reticulum-Associated Degradation (ERAD) Complex to Optimize the Degradation Kinetics of ERAD Substrates. J. Biol. Chem. 286 (19), 16929–16939. 10.1074/jbc.m110.215871 21454652 PMC3089536

[B73] Jalan-Sakrikar N. De Assuncao T. M. Shi G. Aseem S. O. Chi C. Shah V. H. (2019). Proteasomal Degradation of Enhancer of Zeste Homologue 2 in Cholangiocytes Promotes Biliary Fibrosis. Hepatology 70 (5), 1674–1689. 10.1002/hep.30706 31070797 PMC6819212

[B74] Ji C. Kaplowitz N. (2003). Betaine Decreases Hyperhomocysteinemia, Endoplasmic Reticulum Stress, and Liver Injury in Alcohol-Fed Mice. Gastroenterology 124 (5), 1488–1499. 10.1016/s0016-5085(03)00276-2 12730887

[B75] Ji C. Kaplowitz N. Lau M. Y. Kao E. Petrovic L. M. Lee A. S. (2011). Liver-specific Loss of Glucose-Regulated Protein 78 Perturbs the Unfolded Protein Response and Exacerbates a Spectrum of Liver Diseases in Mice. Hepatology 54 (1), 229–239. 10.1002/hep.24368 21503947 PMC3125405

[B76] Ji C. Mehrian-Shai R. Chan C. Hsu Y. H. Kaplowitz N. (2005). Role of CHOP in Hepatic Apoptosis in the Murine Model of Intragastric Ethanol Feeding. Alcohol. Clin. Exp. Res. 29 (8), 1496–1503. 10.1097/01.alc.0000174691.03751.11 16131858 PMC1432051

[B77] Jiang J. Ballinger C. A. Wu Y. Dai Q. Cyr D. M. Hohfeld J. (2001). CHIP Is a U-box-dependent E3 Ubiquitin Ligase: Identification of Hsc70 as a Target for Ubiquitylation. J. Biol. Chem. 276 (46), 42938–42944. 10.1074/jbc.m101968200 11557750

[B78] Jurkin J. Henkel T. Nielsen A. F. Minnich M. Popow J. Kaufmann T. (2014). The Mammalian tRNA Ligase Complex Mediates Splicing of XBP1 mRNA and Controls Antibody Secretion in Plasma Cells. EMBO J. 33 (24), 2922–2936. 10.15252/embj.201490332 25378478 PMC4282640

[B79] Kakazu E. Mauer A. S. Yin M. Malhi H. (2016). Hepatocytes Release Ceramide-Enriched Pro-inflammatory Extracellular Vesicles in an IRE1alpha-dependent Manner. J. Lipid Res. 57 (2), 233–245. 10.1194/jlr.m063412 26621917 PMC4727419

[B80] Kamimoto T. Shoji S. Hidvegi T. Mizushima N. Umebayashi K. Perlmutter D. H. (2006). Intracellular Inclusions Containing Mutant Alpha1-Antitrypsin Z Are Propagated in the Absence of Autophagic Activity. J. Biol. Chem. 281 (7), 4467–4476. 10.1074/jbc.m509409200 16365039

[B81] Kang X. Yang W. Feng D. Jin X. Ma Z. Qian Z. (2017). Cartilage-Specific Autophagy Deficiency Promotes ER Stress and Impairs Chondrogenesis in PERK-ATF4-CHOP-dependent Manner. J. Bone Miner Res. 32 (10), 2128–2141. 10.1002/jbmr.3134 28304100

[B82] Kaushal S. Annamali M. Blomenkamp K. Rudnick D. Halloran D. Brunt E. M. (2010). Rapamycin Reduces Intrahepatic Alpha-1-Antitrypsin Mutant Z Protein Polymers and Liver Injury in a Mouse Model. Exp. Biol. Med. (Maywood). 235 (6), 700–709. 10.1258/ebm.2010.009297 20511674 PMC3763806

[B83] Khaminets A. Heinrich T. Mari M. Grumati P. Huebner A. K. Akutsu M. (2015). Regulation of Endoplasmic Reticulum Turnover by Selective Autophagy. Nature 522 (7556), 354–358. 10.1038/nature14498 26040720

[B84] Kim S. M. Acharya P. Engel J. C. Correia M. A. (2010). Liver Cytochrome P450 3A Ubiquitination *In Vivo* by Gp78/autocrine Motility Factor Receptor and C Terminus of Hsp70-Interacting Protein (CHIP) E3 Ubiquitin Ligases: Physiological and Pharmacological Relevance. J. Biol. Chem. 285 (46), 35866–35877. 10.1074/jbc.m110.167189 20819951 PMC2975210

[B85] Kim S. M. Grenert J. P. Patterson C. Correia M. A. (2016). CHIP(-/-)-Mouse Liver: Adiponectin-AMPK-FOXO-Activation Overrides CYP2E1-Elicited JNK1-Activation, Delaying Onset of NASH: Therapeutic Implications. Sci. Rep. 6, 29423. 10.1038/srep29423 27406999 PMC4942616

[B86] Kim Y. S. Kim S. G. (2020). Endoplasmic Reticulum Stress and Autophagy Dysregulation in Alcoholic and Non-alcoholic Liver Diseases. Clin. Mol. Hepatol. 26 (4), 715–727. 10.3350/cmh.2020.0173 32951410 PMC7641579

[B87] Kleiner D. E. Brunt E. M. Van Natta M. Behling C. Contos M. J. Cummings O. W. (2005). Design and Validation of a Histological Scoring System for Nonalcoholic Fatty Liver Disease. Hepatology 41 (6), 1313–1321. 10.1002/hep.20701 15915461

[B88] Koga H. Kaushik S. Cuervo A. M. (2010). Altered Lipid Content Inhibits Autophagic Vesicular Fusion. FASEB J. 24 (8), 3052–3065. 10.1096/fj.09-144519 20375270 PMC2909278

[B89] Kohno S. Shiozaki Y. Keenan A. L. Miyazaki-Anzai S. Miyazaki M. (2019). An N-Terminal-Truncated Isoform of FAM134B (FAM134B-2) Regulates Starvation-Induced Hepatic Selective ER-Phagy. Life Sci. Alliance 2 (3). 10.26508/lsa.201900340 PMC6526285 31101736

[B90] Koo J. H. Lee H. J. Kim W. Kim S. G. (2016). Endoplasmic Reticulum Stress in Hepatic Stellate Cells Promotes Liver Fibrosis via PERK-Mediated Degradation of HNRNPA1 and Up-Regulation of SMAD2. Gastroenterology 150 (1), 181–193 e8. 10.1053/j.gastro.2015.09.039 26435271

[B91] Kuscuoglu D. Janciauskiene S. Hamesch K. Haybaeck J. Trautwein C. Strnad P. (2018). Liver - Master and Servant of Serum Proteome. J. Hepatol. 69 (2), 512–524. 10.1016/j.jhep.2018.04.018 29709680

[B92] Kwon D. Kim S. M. Correia M. A. (2020). Cytochrome P450 Endoplasmic Reticulum-Associated Degradation (ERAD): Therapeutic and Pathophysiological Implications. Acta Pharm. Sin B 10 (1), 42–60. 10.1016/j.apsb.2019.11.002 31993306 PMC6976991

[B93] Kwon D. Kim S. M. Jacob P. Liu Y. 3rd Correia M. A. (2019). Induction via Functional Protein Stabilization of Hepatic Cytochromes P450 upon gp78/Autocrine Motility Factor Receptor (AMFR) Ubiquitin E3-Ligase Genetic Ablation in Mice: Therapeutic and Toxicological Relevance. Mol. Pharmacol. 96 (5), 641–654. 10.1124/mol.119.117069 31492698 PMC6790065

[B94] Li H. Meng Q. Xiao F. Chen S. Du Y. Yu J. (2011). ATF4 Deficiency Protects Mice from High-Carbohydrate-Diet-Induced Liver Steatosis. Biochem. J. 438 (2), 283–289. 10.1042/bj20110263 21644928

[B95] Li K. Xiao Y. Yu J. Xia T. Liu B. Guo Y. (2016). Liver-specific Gene Inactivation of the Transcription Factor ATF4 Alleviates Alcoholic Liver Steatosis in Mice. J. Biol. Chem. 291 (35), 18536–18546. 10.1074/jbc.m116.726836 27405764 PMC5000098

[B96] Li K. Zhang K. Wang H. Wu Y. Chen N. Chen J. (2021). Hrd1-mediated ACLY Ubiquitination Alleviate NAFLD in Db/db Mice. Metabolism 114, 154349. 10.1016/j.metabol.2020.154349 32888949

[B97] Liang J. R. Lingeman E. Ahmed S. Corn J. E. (2018). Atlastins Remodel the Endoplasmic Reticulum for Selective Autophagy. J. Cell Biol 217 (10), 3354–3367. 10.1083/jcb.201804185 30143524 PMC6168278

[B98] Liang J. R. Lingeman E. Luong T. Ahmed S. Muhar M. Nguyen T. (2020). A Genome-wide ER-Phagy Screen Highlights Key Roles of Mitochondrial Metabolism and ER-Resident UFMylation. Cell 180 (6), 1160–1177. 10.1016/j.cell.2020.02.017 32160526 PMC7197389

[B99] Lilley B. N. Ploegh H. L. (2005). Multiprotein Complexes that Link Dislocation, Ubiquitination, and Extraction of Misfolded Proteins from the Endoplasmic Reticulum Membrane. Proc. Natl. Acad. Sci. U S A. 102 (40), 14296–14301. 10.1073/pnas.0505014102 16186509 PMC1242303

[B100] Lin C. W. Zhang H. Li M. Xiong X. Chen X. Chen X. (2013). Pharmacological Promotion of Autophagy Alleviates Steatosis and Injury in Alcoholic and Non-alcoholic Fatty Liver Conditions in Mice. J. Hepatol. 58 (5), 993–999. 10.1016/j.jhep.2013.01.011 23339953 PMC3634371

[B101] Liu H. Y. Han J. Cao S. Y. Hong T. Zhuo D. Shi J. (2009). Hepatic Autophagy Is Suppressed in the Presence of Insulin Resistance and Hyperinsulinemia: Inhibition of FoxO1-dependent Expression of Key Autophagy Genes by Insulin. J. Biol. Chem. 284 (45), 31484–31492. 10.1074/jbc.m109.033936 19758991 PMC2781544

[B102] Liu P. Bartz R. Zehmer J. K. Ying Y. S. Zhu M. Serrero G. (2007). Rab-regulated Interaction of Early Endosomes with Lipid Droplets. Biochim. Biophys. Acta 1773 (6), 784–793. 10.1016/j.bbamcr.2007.02.004 17395284 PMC2676670

[B103] Liu Q. Yang X. Long G. Hu Y. Gu Z. Boisclair Y. R. (2020). ERAD Deficiency Promotes Mitochondrial Dysfunction and Transcriptional Rewiring in Human Hepatic Cells. J. Biol. Chem. 295 (49), 16743–16753. 10.1074/jbc.ra120.013987 32978261 PMC7864069

[B104] Liu T. F. Tang J. J. Li P. S. Shen Y. Li J. G. Miao H. H. (2012). Ablation of Gp78 in Liver Improves Hyperlipidemia and Insulin Resistance by Inhibiting SREBP to Decrease Lipid Biosynthesis. Cell Metab 16 (2), 213–225. 10.1016/j.cmet.2012.06.014 22863805

[B105] Liu W. Baker S. S. Baker R. D. Nowak N. J. Zhu L. (2011). Upregulation of Hemoglobin Expression by Oxidative Stress in Hepatocytes and its Implication in Nonalcoholic Steatohepatitis. PLoS One 6 (9), e24363. 10.1371/journal.pone.0024363 21931690 PMC3171444

[B106] Liu Z. Li C. Kang N. Malhi H. Shah V. H. Maiers J. L. (2019). Transforming Growth Factor Beta (TGFbeta) Cross-Talk with the Unfolded Protein Response Is Critical for Hepatic Stellate Cell Activation. J. Biol. Chem. 294 (9), 3137–3151. 10.1074/jbc.ra118.005761 30610118 PMC6398135

[B107] Loi M. Raimondi A. Morone D. Molinari M. (2019). ESCRT-III-driven Piecemeal Micro-ER-phagy Remodels the ER during Recovery from ER Stress. Nat. Commun. 10 (1), 5058. 10.1038/s41467-019-12991-z 31699981 PMC6838186

[B108] Lu Y. Cederbaum A. I. (2015). Autophagy Protects against CYP2E1/Chronic Ethanol-Induced Hepatotoxicity. Biomolecules 5 (4), 2659–2674. 10.3390/biom5042659 26501338 PMC4693252

[B109] Lu Y. Cederbaum A. I. (2018). Cytochrome P450s and Alcoholic Liver Disease. Curr. Pharm. Des. 24 (14), 1502–1517. 10.2174/1381612824666180410091511 29637855 PMC6053342

[B110] Lucantoni F. Martinez-Cerezuela A. Gruevska A. Moragrega A. B. Victor V. M. Esplugues J. V. (2021). Understanding the Implication of Autophagy in the Activation of Hepatic Stellate Cells in Liver Fibrosis: Are We There yet? J. Pathol. 254 (3), 216–228. 10.1002/path.5678 33834482

[B111] Ma X. Parson C. Ding W. X. (2018). Regulation of the Homeostasis of Hepatic Endoplasmic Reticulum and Cytochrome P450 Enzymes by Autophagy. Liver Res. 2 (3), 138–145. 10.1016/j.livres.2018.08.004 31807367 PMC6894516

[B112] Maiers J. L. Malhi H. (2019). Endoplasmic Reticulum Stress in Metabolic Liver Diseases and Hepatic Fibrosis. Semin. Liver Dis. 39 (2), 235–248. 10.1055/s-0039-1681032 30912096 PMC6530577

[B113] Malhi H. Kropp E. M. Clavo V. F. Kobrossi C. R. Han J. Mauer A. S. (2013). C/EBP Homologous Protein-Induced Macrophage Apoptosis Protects Mice from Steatohepatitis. J. Biol. Chem. 288 (26), 18624–18642. 10.1074/jbc.m112.442954 23720735 PMC3696637

[B114] Manne V. Kowdley K. V. (2020). Alpha1-Antitrypsin Deficiency: A Cause of Chronic Liver Disease. Clin. Liver Dis. 24 (3), 483–492. 10.1016/j.cld.2020.04.010 32620284

[B115] Mas V. R. Maluf D. G. Archer K. J. Yanek K. Kong X. Kulik L. (2009). Genes Involved in Viral Carcinogenesis and Tumor Initiation in Hepatitis C Virus-Induced Hepatocellular Carcinoma. Mol. Med. 15 (3-4), 85–94. 10.2119/molmed.2008.00110 19098997 PMC2605622

[B116] Masouminia M. Samadzadeh S. Ebaee A. French B. A. Tillman B. French S. W. (2016). Alcoholic Steatohepatitis (ASH) Causes More UPR-ER Stress Than Non-alcoholic Steatohepatitis (NASH). Exp. Mol. Pathol. 101 (2), 201–206. 10.1016/j.yexmp.2016.08.002 27587085 PMC5115941

[B117] Miao H. Jiang W. Ge L. Li B. Song B. (2010). Tetra-glutamic Acid Residues Adjacent to Lys248 in HMG-CoA Reductase Are Critical for the Ubiquitination Mediated by Gp78 and UBE2G2. Acta Biochim. Biophys. Sin (Shanghai). 42 (5), 303–310. 10.1093/abbs/gmq022 20458442

[B118] Molinari M. (2021). ER-phagy Responses in Yeast, Plants, and Mammalian Cells and Their Crosstalk with UPR and ERAD. Dev. Cell 56 (7), 949–966. 10.1016/j.devcel.2021.03.005 33765438

[B119] Moore K. A. Hollien J. (2012). The Unfolded Protein Response in Secretory Cell Function. Annu. Rev. Genet. 46, 165–183. 10.1146/annurev-genet-110711-155644 22934644

[B120] Morishima Y. Peng H. M. Lin H. L. Hollenberg P. F. Sunahara R. K. Osawa Y. (2005). Regulation of Cytochrome P450 2E1 by Heat Shock Protein 90-dependent Stabilization and CHIP-dependent Proteasomal Degradation. Biochemistry 44 (49), 16333–16340. 10.1021/bi0515570 16331994

[B121] Murata S. Minami Y. Minami M. Chiba T. Tanaka K. (2001). CHIP Is a Chaperone-dependent E3 Ligase that Ubiquitylates Unfolded Protein. EMBO Rep. 2 (12), 1133–1138. 10.1093/embo-reports/kve246 11743028 PMC1084164

[B122] Murphy S. K. Yang H. Moylan C. A. Pang H. Dellinger A. Abdelmalek M. F. (2013). Relationship between Methylome and Transcriptome in Patients with Nonalcoholic Fatty Liver Disease. Gastroenterology 145 (5), 1076–1087. 10.1053/j.gastro.2013.07.047 23916847 PMC3805742

[B123] Nagy L. E. Ding W. X. Cresci G. Saikia P. Shah V. H. (2016). Linking Pathogenic Mechanisms of Alcoholic Liver Disease with Clinical Phenotypes. Gastroenterology 150 (8), 1756–1768. 10.1053/j.gastro.2016.02.035 26919968 PMC4887335

[B124] Narayanan P. Mistry P. K. (2020). Update on Alpha-1 Antitrypsin Deficiency in Liver Disease. Clin. Liver Dis. (Hoboken). 15 (6), 228–235. 10.1002/cld.896 32617155 PMC7326637

[B125] Ninagawa S. George G. Mori K. (2021). Mechanisms of Productive Folding and Endoplasmic Reticulum-Associated Degradation of Glycoproteins and Non-glycoproteins. Biochim. Biophys. Acta Gen. Subj 1865 (3), 129812. 10.1016/j.bbagen.2020.129812 33316349

[B126] Nthiga T. M. Kumar Shrestha B. Sjottem E. Bruun J. A. Bowitz Larsen K. Bhujabal Z. (2020). CALCOCO1 Acts with VAMP-Associated Proteins to Mediate ER-Phagy. EMBO J. 39 (15), e103649. 10.15252/embj.2019103649 32525583 PMC7396842

[B127] Oda Y. Okada T. Yoshida H. Kaufman R. J. Nagata K. Mori K. (2006). Derlin-2 and Derlin-3 Are Regulated by the Mammalian Unfolded Protein Response and Are Required for ER-Associated Degradation. J. Cell Biol 172 (3), 383–393. 10.1083/jcb.200507057 16449189 PMC2063648

[B128] Olzmann J. A. Kopito R. R. Christianson J. C. (2013). The Mammalian Endoplasmic Reticulum-Associated Degradation System. Cold Spring Harb Perspect. Biol. 5 (9). 10.1101/cshperspect.a013185 PMC3753711 23232094

[B129] Omari S. Makareeva E. Roberts-Pilgrim A. Mirigian L. Jarnik M. Ott C. (2018). Noncanonical Autophagy at ER Exit Sites Regulates Procollagen Turnover. Proc. Natl. Acad. Sci. U S A. 115 (43), E10099–E108. 10.1073/pnas.1814552115 30287488 PMC6205486

[B130] Oyadomari S. Harding H. P. Zhang Y. Oyadomari M. Ron D. (2008). Dephosphorylation of Translation Initiation Factor 2alpha Enhances Glucose Tolerance and Attenuates Hepatosteatosis in Mice. Cell Metab 7 (6), 520–532. 10.1016/j.cmet.2008.04.011 18522833 PMC2474721

[B131] Park S. M. Kang T. I. So J. S. (2021). Roles of XBP1s in Transcriptional Regulation of Target Genes. Biomedicines 9 (7). 10.3390/biomedicines9070791 PMC8301375 34356855

[B132] Pastore N. Ballabio A. Brunetti-Pierri N. (2013). Autophagy Master Regulator TFEB Induces Clearance of Toxic SERPINA1/alpha-1-Antitrypsin Polymers. Autophagy 9 (7), 1094–1096. 10.4161/auto.24469 23584152 PMC3722318

[B133] Pastore N. Nusco E. Vanikova J. Sepe R. M. Vetrini F. McDonagh A. (2012). Sustained Reduction of Hyperbilirubinemia in Gunn Rats after Adeno-Associated Virus-Mediated Gene Transfer of Bilirubin UDP-Glucuronosyltransferase Isozyme 1A1 to Skeletal Muscle. Hum. Gene Ther. 23 (10), 1082–1089. 10.1089/hum.2012.018 22765254

[B134] Patel D. McAllister S. L. Teckman J. H. (2021). Alpha-1 Antitrypsin Deficiency Liver Disease. Transl Gastroenterol. Hepatol. 6, 23. 10.21037/tgh.2020.02.23 33824927 PMC7829072

[B135] Penke L. R. K. Speth J. Wettlaufer S. Draijer C. Peters-Golden M. (2021). Bortezomib Inhibits Lung Fibrosis and Fibroblast Activation without Proteasome Inhibition. Am. J. Respir. Cell Mol Biol. [Epub ahead of print]. 10.1165/rcmb.2021-0112oc PMC8803353 34236953

[B136] Perlmutter D. H. (2006). The Role of Autophagy in Alpha-1-Antitrypsin Deficiency: a Specific Cellular Response in Genetic Diseases Associated with Aggregation-Prone Proteins. Autophagy 2 (4), 258–263. 10.4161/auto.2882 16874089

[B137] Petersen K. F. Dufour S. Hariri A. Nelson-Williams C. Foo J. N. Zhang X. M. (2010). Apolipoprotein C3 Gene Variants in Nonalcoholic Fatty Liver Disease. N. Engl. J. Med. 362 (12), 1082–1089. 10.1056/nejmoa0907295 20335584 PMC2976042

[B138] Pfaffenbach K. T. Gentile C. L. Nivala A. M. Wang D. Wei Y. Pagliassotti M. J. (2010). Linking Endoplasmic Reticulum Stress to Cell Death in Hepatocytes: Roles of C/EBP Homologous Protein and Chemical Chaperones in Palmitate-Mediated Cell Death. Am. J. Physiol. Endocrinol. Metab. 298 (5), E1027–E1035. 10.1152/ajpendo.00642.2009 20159858 PMC2867372

[B139] Qu D. Teckman J. H. Omura S. Perlmutter D. H. (1996). Degradation of a Mutant Secretory Protein, Alpha1-Antitrypsin Z, in the Endoplasmic Reticulum Requires Proteasome Activity. J. Biol. Chem. 271 (37), 22791–22795. 10.1074/jbc.271.37.22791 8798455

[B140] Rahman K. Liu Y. Kumar P. Smith T. Thorn N. E. Farris A. B. (2016). C/EBP Homologous Protein Modulates Liraglutide-Mediated Attenuation of Non-alcoholic Steatohepatitis. Lab. Invest. 96 (8), 895–908. 10.1038/labinvest.2016.61 27239734 PMC4965279

[B141] Ramos V. M. Kowaltowski A. J. Kakimoto P. A. (2021). Autophagy in Hepatic Steatosis: A Structured Review. Front Cell Dev Biol 9, 657389. 10.3389/fcell.2021.657389 33937257 PMC8081956

[B142] Rutkowski D. T. Hegde R. S. (2010). Regulation of Basal Cellular Physiology by the Homeostatic Unfolded Protein Response. J. Cell Biol 189 (5), 783–794. 10.1083/jcb.201003138 20513765 PMC2878945

[B143] Rutkowski D. T. Kaufman R. J. (2004). A Trip to the ER: Coping with Stress. Trends Cell Biol 14 (1), 20–28. 10.1016/j.tcb.2003.11.001 14729177

[B144] Rutkowski D. T. Wu J. Back S. H. Callaghan M. U. Ferris S. P. Iqbal J. (2008). UPR Pathways Combine to Prevent Hepatic Steatosis Caused by ER Stress-Mediated Suppression of Transcriptional Master Regulators. Dev. Cell 15 (6), 829–840. 10.1016/j.devcel.2008.10.015 19081072 PMC2923556

[B145] Sarphie G. D'Souza N. B. Van Thiel D. H. Hill D. McClain C. J. Deaciuc I. V. (1997). Dose- and Time-dependent Effects of Ethanol on Functional and Structural Aspects of the Liver Sinusoid in the Mouse. Alcohol. Clin. Exp. Res. 21 (6), 1128–1136. 10.1111/j.1530-0277.1997.tb04263.x 9309327

[B146] Schulze R. J. Schott M. B. Casey C. A. Tuma P. L. McNiven M. A. (2019). The Cell Biology of the Hepatocyte: A Membrane Trafficking Machine. J. Cell Biol 218 (7), 2096–2112. 10.1083/jcb.201903090 31201265 PMC6605791

[B147] Schwarz D. S. Blower M. D. (2016). The Endoplasmic Reticulum: Structure, Function and Response to Cellular Signaling. Cell Mol Life Sci 73 (1), 79–94. 10.1007/s00018-015-2052-6 26433683 PMC4700099

[B148] Shan B. Wang X. Wu Y. Xu C. Xia Z. Dai J. (2017). The Metabolic ER Stress Sensor IRE1alpha Suppresses Alternative Activation of Macrophages and Impairs Energy Expenditure in Obesity. Nat. Immunol. 18 (5), 519–529. 10.1038/ni.3709 28346409

[B149] Shen J. Chen X. Hendershot L. Prywes R. (2002). ER Stress Regulation of ATF6 Localization by Dissociation of BiP/GRP78 Binding and Unmasking of Golgi Localization Signals. Dev. Cell 3 (1), 99–111. 10.1016/s1534-5807(02)00203-4 12110171

[B150] Shen Y. Ballar P. Fang S. (2006). Ubiquitin Ligase Gp78 Increases Solubility and Facilitates Degradation of the Z Variant of Alpha-1-Antitrypsin. Biochem. Biophys. Res. Commun. 349 (4), 1285–1293. 10.1016/j.bbrc.2006.08.173 16979136

[B151] Silverman E. K. Sandhaus R. A. (2009). Clinical Practice. Alpha1-Antitrypsin Deficiency. N. Engl. J. Med. 360 (26), 2749–2757. 10.1056/nejmcp0900449 19553648

[B152] Simon Y. Kessler S. M. Gemperlein K. Bohle R. M. Muller R. Haybaeck J. (2014). Elevated Free Cholesterol in a P62 Overexpression Model of Non-alcoholic Steatohepatitis. World J. Gastroenterol. 20 (47), 17839–17850. 10.3748/wjg.v20.i47.17839 25548482 PMC4273134

[B153] Singh R. Kaushik S. Wang Y. Xiang Y. Novak I. Komatsu M. (2009). Autophagy Regulates Lipid Metabolism. Nature 458 (7242), 1131–1135. 10.1038/nature07976 19339967 PMC2676208

[B154] Smith M. D. Harley M. E. Kemp A. J. Wills J. Lee M. Arends M. (2018). CCPG1 Is a Non-canonical Autophagy Cargo Receptor Essential for ER-Phagy and Pancreatic ER Proteostasis. Dev. Cell 44 (2), 217–232. 10.1016/j.devcel.2017.11.024 29290589 PMC5791736

[B155] Song B. J. Abdelmegeed M. A. Cho Y. E. Akbar M. Rhim J. S. Song M. K. (2019). Contributing Roles of CYP2E1 and Other Cytochrome P450 Isoforms in Alcohol-Related Tissue Injury and Carcinogenesis. Adv. Exp. Med. Biol. [Epub ahead of print] 1164, 73–87. 10.1007/978-3-030-22254-3_6 31576541

[B156] Song J. Liu Y. Wan J. Zhao G. N. Wang J. C. Dai Z. (2021). SIMPLE Is an Endosomal Regulator that Protects against Non-alcoholic Fatty Liver Disease by Targeting the Lysosomal Degradation of EGFR. Hepatology. 10.1002/hep.32075 34320238

[B157] Song Q. Chen Y. Wang J. Hao L. Huang C. Griffiths A. (2020). ER Stress-Induced Upregulation of NNMT Contributes to Alcohol-Related Fatty Liver Development. J. Hepatol. 73 (4), 783–793. 10.1016/j.jhep.2020.04.038 32389809 PMC8301603

[B158] Starmann J. Falth M. Spindelbock W. Lanz K. L. Lackner C. Zatloukal K. (2012). Gene Expression Profiling Unravels Cancer-Related Hepatic Molecular Signatures in Steatohepatitis but Not in Steatosis. PLoS One 7 (10), e46584. 10.1371/journal.pone.0046584 23071592 PMC3468618

[B159] Stephani M. Picchianti L. Gajic A. Beveridge R. Skarwan E. Sanchez de Medina Hernandez V. (2020). A Cross-Kingdom Conserved ER-Phagy Receptor Maintains Endoplasmic Reticulum Homeostasis during Stress. Elife 9. 10.7554/eLife.58396 PMC7515635 32851973

[B160] Strong A. Ding Q. Edmondson A. C. Millar J. S. Sachs K. V. Li X. (2012). Hepatic Sortilin Regulates Both Apolipoprotein B Secretion and LDL Catabolism. J. Clin. Invest. 122 (8), 2807–2816. 10.1172/jci63563 22751103 PMC3408750

[B161] Sun M. Tan L. Hu M. (2021). The Role of Autophagy in Hepatic Fibrosis. Am. J. Transl Res. 13 (6), 5747–5757. 34306323 PMC8290830

[B162] Sun Z. Brodsky J. L. (2019). Protein Quality Control in the Secretory Pathway. J. Cell Biol 218 (10), 3171–3187. 10.1083/jcb.201906047 31537714 PMC6781448

[B163] Tan J. M. E. Cook E. C. L. van den Berg M. Scheij S. Zelcer N. Loregger A. (2019). Differential Use of E2 Ubiquitin Conjugating Enzymes for Regulated Degradation of the Rate-Limiting Enzymes HMGCR and SQLE in Cholesterol Biosynthesis. Atherosclerosis 281, 137–142. 10.1016/j.atherosclerosis.2018.12.008 30658189

[B164] Tang Y. Blomenkamp K. S. Fickert P. Trauner M. Teckman J. H. (2018). NorUDCA Promotes Degradation of Alpha1-Antitrypsin Mutant Z Protein by Inducing Autophagy through AMPK/ULK1 Pathway. PLoS One 13 (8), e0200897. 10.1371/journal.pone.0200897 30067827 PMC6070232

[B165] Tang Y. Fickert P. Trauner M. Marcus N. Blomenkamp K. Teckman J. (2016). Autophagy Induced by Exogenous Bile Acids Is Therapeutic in a Model of Alpha-1-AT Deficiency Liver Disease. Am. J. Physiol. Gastrointest. Liver Physiol. 311 (1), G156–G165. 10.1152/ajpgi.00143.2015 27102560

[B166] Teckman J. H. Perlmutter D. H. (2000). Retention of Mutant Alpha(1)-Antitrypsin Z in Endoplasmic Reticulum Is Associated with an Autophagic Response. Am. J. Physiol. Gastrointest. Liver Physiol. 279 (5), G961–G974. 10.1152/ajpgi.2000.279.5.g961 11052993

[B167] Teckman J. H. Qu D. Perlmutter D. H. (1996). Molecular Pathogenesis of Liver Disease in Alpha1-Antitrypsin Deficiency. Hepatology 24 (6), 1504–1516. 10.1053/jhep.1996.v24.ajhep0241504 8938188

[B168] Teschke R. (2018). Alcoholic Liver Disease: Alcohol Metabolism, Cascade of Molecular Mechanisms, Cellular Targets, and Clinical Aspects. Biomedicines 6 (4). 10.3390/biomedicines6040106 PMC6316574 30424581

[B169] Tirosh B. Iwakoshi N. N. Glimcher L. H. Ploegh H. L. (2006). Rapid Turnover of Unspliced Xbp-1 as a Factor that Modulates the Unfolded Protein Response. J. Biol. Chem. 281 (9), 5852–5860. 10.1074/jbc.m509061200 16332684

[B170] Toriguchi K. Hatano E. Tanabe K. Takemoto K. Nakamura K. Koyama Y. (2014). Attenuation of Steatohepatitis, Fibrosis, and Carcinogenesis in Mice Fed a Methionine-Choline Deficient Diet by CCAAT/enhancer-binding Protein Homologous Protein Deficiency. J. Gastroenterol. Hepatol. 29 (5), 1109–1118. 10.1111/jgh.12481 24329600

[B171] Trepo E. Goossens N. Fujiwara N. Song W. M. Colaprico A. Marot A. (2018). Combination of Gene Expression Signature and Model for End-Stage Liver Disease Score Predicts Survival of Patients with Severe Alcoholic Hepatitis. Gastroenterology 154 (4), 965–975. 10.1053/j.gastro.2017.10.048 29158192 PMC5847453

[B172] Tsedensodnom O. Vacaru A. M. Howarth D. L. Yin C. Sadler K. C. (2013). Ethanol Metabolism and Oxidative Stress Are Required for Unfolded Protein Response Activation and Steatosis in Zebrafish with Alcoholic Liver Disease. Dis. Model. Mech. 6 (5), 1213–1226. 10.1242/dmm.012195 23798569 PMC3759341

[B173] Vembar S. S. Brodsky J. L. (2008). One Step at a Time: Endoplasmic Reticulum-Associated Degradation. Nat. Rev. Mol. Cell Biol 9 (12), 944–957. 10.1038/nrm2546 19002207 PMC2654601

[B174] Vos D. Y. van de Sluis B. (2021). Function of the Endolysosomal Network in Cholesterol Homeostasis and Metabolic-Associated Fatty Liver Disease (MAFLD). Mol. Metab. 50, 101146. 10.1016/j.molmet.2020.101146 33348067 PMC8324686

[B175] Wang B. Yang H. Fan Y. Yang Y. Cao W. Jia Y. (2017). 3-Methyladenine Ameliorates Liver Fibrosis through Autophagy Regulated by the NF-kappaB Signaling Pathways on Hepatic Stellate Cell. Oncotarget 8 (64), 107603–107611. 10.18632/oncotarget.22539 29296191 PMC5746093

[B176] Wang H. Li Q. Shen Y. Sun A. Zhu X. Fang S. (2011). The Ubiquitin Ligase Hrd1 Promotes Degradation of the Z Variant Alpha 1-antitrypsin and Increases its Solubility. Mol. Cell Biochem 346 (1-2), 137–145. 10.1007/s11010-010-0600-9 20886262

[B177] Wang J. M. Qiu Y. Yang Z. Kim H. Qian Q. Sun Q. (2018). IRE1alpha Prevents Hepatic Steatosis by Processing and Promoting the Degradation of Select microRNAs. Sci. Signal. 11 (530). 10.1126/scisignal.aao4617 PMC6075656 29764990

[B178] Wang Y. Cobanoglu M. C. Li J. Hidvegi T. Hale P. Ewing M. (2019). An Analog of Glibenclamide Selectively Enhances Autophagic Degradation of Misfolded Alpha1-Antitrypsin Z. PLoS One 14 (1), e0209748. 10.1371/journal.pone.0209748 30673724 PMC6343872

[B179] Wang Y. Shen J. Arenzana N. Tirasophon W. Kaufman R. J. Prywes R. (2000). Activation of ATF6 and an ATF6 DNA Binding Site by the Endoplasmic Reticulum Stress Response. J. Biol. Chem. 275 (35), 27013–27020. 10.1016/s0021-9258(19)61473-0 10856300

[B180] Wang Y. Shi M. Fu H. Xu H. Wei J. Wang T. (2010). Mammalian Target of the Rapamycin Pathway Is Involved in Non-alcoholic Fatty Liver Disease. Mol. Med. Rep. 3 (6), 909–915. 10.3892/mmr.2010.365 21472332

[B181] Wei J. Yuan Y. Chen L. Xu Y. Zhang Y. Wang Y. (2018). ER-associated Ubiquitin Ligase HRD1 Programs Liver Metabolism by Targeting Multiple Metabolic Enzymes. Nat. Commun. 9 (1), 3659. 10.1038/s41467-018-06091-7 30201971 PMC6131148

[B182] Werstuck G. H. Lentz S. R. Dayal S. Hossain G. S. Sood S. K. Shi Y. Y. (2001). Homocysteine-induced Endoplasmic Reticulum Stress Causes Dysregulation of the Cholesterol and Triglyceride Biosynthetic Pathways. J. Clin. Invest. 107 (10), 1263–1273. 10.1172/jci11596 11375416 PMC209295

[B183] Williams J. A. Ding W. X. (2020). Role of Autophagy in Alcohol and Drug-Induced Liver Injury. Food Chem. Toxicol. 136, 111075. 10.1016/j.fct.2019.111075 31877367 PMC6947668

[B184] Wu D. Cederbaum A. I. (1996). Ethanol Cytotoxicity to a Transfected HepG2 Cell Line Expressing Human Cytochrome P4502E1. J. Biol. Chem. 271 (39), 23914–23919. 10.1074/jbc.271.39.23914 8798623

[B185] Wu W. K. K. Zhang L. Chan M. T. V. (2018). Autophagy, NAFLD and NAFLD-Related HCC. Adv. Exp. Med. Biol. 1061, 127–138. 10.1007/978-981-10-8684-7_10 29956211

[B186] Xia S. W. Wang Z. M. Sun S. M. Su Y. Li Z. H. Shao J. J. (2020). Endoplasmic Reticulum Stress and Protein Degradation in Chronic Liver Disease. Pharmacol. Res. 161, 105218. 10.1016/j.phrs.2020.105218 33007418

[B187] Xiao G. Zhang T. Yu S. Lee S. Calabuig-Navarro V. Yamauchi J. (2013). ATF4 Protein Deficiency Protects against High Fructose-Induced Hypertriglyceridemia in Mice. J. Biol. Chem. 288 (35), 25350–25361. 10.1074/jbc.m113.470526 23888053 PMC3757199

[B188] Xie Z. Y. Xiao Z. H. Wang F. F. (2018). Inhibition of Autophagy Reverses Alcohol-Induced Hepatic Stellate Cells Activation through Activation of Nrf2-Keap1-ARE Signaling Pathway. Biochimie 147, 55–62. 10.1016/j.biochi.2017.12.013 29305174

[B189] Xue F. Lu J. Buchl S. C. Sun L. Shah V. H. Malhi H. (2021). Coordinated Signaling of Activating Transcription Factor 6alpha and Inositol-Requiring Enzyme 1alpha Regulates Hepatic Stellate Cell-Mediated Fibrogenesis in Mice. Am. J. Physiol. Gastrointest. Liver Physiol. 320 (5), G864–G79. 10.1152/ajpgi.00453.2020 33728997 PMC8202196

[B190] Yamamoto K. Sato T. Matsui T. Sato M. Okada T. Yoshida H. (2007). Transcriptional Induction of Mammalian ER Quality Control Proteins Is Mediated by Single or Combined Action of ATF6alpha and XBP1. Dev. Cell 13 (3), 365–376. 10.1016/j.devcel.2007.07.018 17765680

[B191] Yamamoto K. Takahara K. Oyadomari S. Okada T. Sato T. Harada A. (2010). Induction of Liver Steatosis and Lipid Droplet Formation in ATF6alpha-Knockout Mice Burdened with Pharmacological Endoplasmic Reticulum Stress. Mol. Biol. Cell 21 (17), 2975–2986. 10.1091/mbc.e09-02-0133 20631254 PMC2929991

[B192] Yamamura T. Ohsaki Y. Suzuki M. Shinohara Y. Tatematsu T. Cheng J. (2014). Inhibition of Niemann-pick-type C1-Like1 by Ezetimibe Activates Autophagy in Human Hepatocytes and Reduces Mutant Alpha1-Antitrypsin Z Deposition. Hepatology 59 (4), 1591–1599. 10.1002/hep.26930 24214142

[B193] Yang H. Ni H. M. Guo F. Ding Y. Shi Y. H. Lahiri P. (2016). Sequestosome 1/p62 Protein Is Associated with Autophagic Removal of Excess Hepatic Endoplasmic Reticulum in Mice. J. Biol. Chem. 291 (36), 18663–18674. 10.1074/jbc.m116.739821 27325701 PMC5009243

[B194] Yang L. Li P. Fu S. Calay E. S. Hotamisligil G. S. (2010). Defective Hepatic Autophagy in Obesity Promotes ER Stress and Causes Insulin Resistance. Cell Metab 11 (6), 467–478. 10.1016/j.cmet.2010.04.005 20519119 PMC2881480

[B195] Yang Q. Chen X. Zhang Y. Hu S. Hu F. Huang Y. (2021). The E3 Ubiquitin Ligase RNF5 Ameliorates Nonalcoholic Steatohepatitis via Ubiquitin-Mediated Degradation of HRD1. Hepatology. 10.1002/hep.32061

[B196] Yang Y. Sangwung P. Kondo R. Jung Y. McConnell M. J. Jeong J. (2021). Alcohol-induced Hsp90 Acetylation Is a Novel Driver of Liver Sinusoidal Endothelial Dysfunction and Alcohol-Related Liver Disease. J. Hepatol. 75 (2), 377–386. 10.1016/j.jhep.2021.02.028 33675874 PMC8292196

[B197] Ye Y. Shibata Y. Yun C. Ron D. Rapoport T. A. (2004). A Membrane Protein Complex Mediates Retro-Translocation from the ER Lumen into the Cytosol. Nature 429 (6994), 841–847. 10.1038/nature02656 15215856

[B198] Yoshida H. Haze K. Yanagi H. Yura T. Mori K. (1998). Identification of the Cis-Acting Endoplasmic Reticulum Stress Response Element Responsible for Transcriptional Induction of Mammalian Glucose-Regulated Proteins. Involvement of Basic Leucine Zipper Transcription Factors. J. Biol. Chem. 273 (50), 33741–33749. 10.1074/jbc.273.50.33741 9837962

[B199] Younossi Z. Anstee Q. M. Marietti M. Hardy T. Henry L. Eslam M. (2018). Global burden of NAFLD and NASH: Trends, Predictions, Risk Factors and Prevention. Nat. Rev. Gastroenterol. Hepatol. 15 (1), 11–20. 10.1038/nrgastro.2017.109 28930295

[B200] Younossi Z. M. Koenig A. B. Abdelatif D. Fazel Y. Henry L. Wymer M. (2016). Global Epidemiology of Nonalcoholic Fatty Liver Disease-Meta-Analytic Assessment of Prevalence, Incidence, and Outcomes. Hepatology 64 (1), 73–84. 10.1002/hep.28431 26707365

[B201] Zeigerer A. Gilleron J. Bogorad R. L. Marsico G. Nonaka H. Seifert S. (2012). Rab5 Is Necessary for the Biogenesis of the Endolysosomal System *In Vivo* . Nature 485 (7399), 465–470. 10.1038/nature11133 22622570

[B202] Zhang K. Wang S. Malhotra J. Hassler J. R. Back S. H. Wang G. (2011). The Unfolded Protein Response Transducer IRE1alpha Prevents ER Stress-Induced Hepatic Steatosis. EMBO J. 30 (7), 1357–1375. 10.1038/emboj.2011.52 21407177 PMC3094110

[B203] Zhang T. Kho D. H. Wang Y. Harazono Y. Nakajima K. Xie Y. (2015). Gp78, an E3 Ubiquitin Ligase Acts as a Gatekeeper Suppressing Nonalcoholic Steatohepatitis (NASH) and Liver Cancer. PLoS One 10 (3), e0118448. 10.1371/journal.pone.0118448 25789613 PMC4366401

[B204] Zheng W. Xie W. Yin D. Luo R. Liu M. Guo F. (2019). ATG5 and ATG7 Induced Autophagy Interplays with UPR via PERK Signaling. Cell Commun Signal 17 (1), 42. 10.1186/s12964-019-0353-3 31060556 PMC6503447

[B205] Zhou J. Cheng H. Wang Z. Chen H. Suo C. Zhang H. (2019). Bortezomib Attenuates Renal Interstitial Fibrosis in Kidney Transplantation via Regulating the EMT Induced by TNF-Alpha-Smurf1-Akt-mTOR-P70s6k Pathway. J. Cell Mol Med [Epub ahead of print] 23 (8), 5390–5402. 10.1111/jcmm.14420 31140729 PMC6653435

[B206] Zhou J. C. Wang J. L. Ren H. Z. Shi X. L. (2021). Autophagy Plays a Double-Edged Sword Role in Liver Diseases. J. Physiol. Biochem. 10.1007/s13105-021-00844-7 PMC8873123 34657993

[B207] Zhu J. Wu J. Frizell E. Liu S. L. Bashey R. Rubin R. (1999). Rapamycin Inhibits Hepatic Stellate Cell Proliferation *In Vitro* and Limits Fibrogenesis in an *In Vivo* Model of Liver Fibrosis. Gastroenterology 117 (5), 1198–1204. 10.1016/s0016-5085(99)70406-3 10535884

